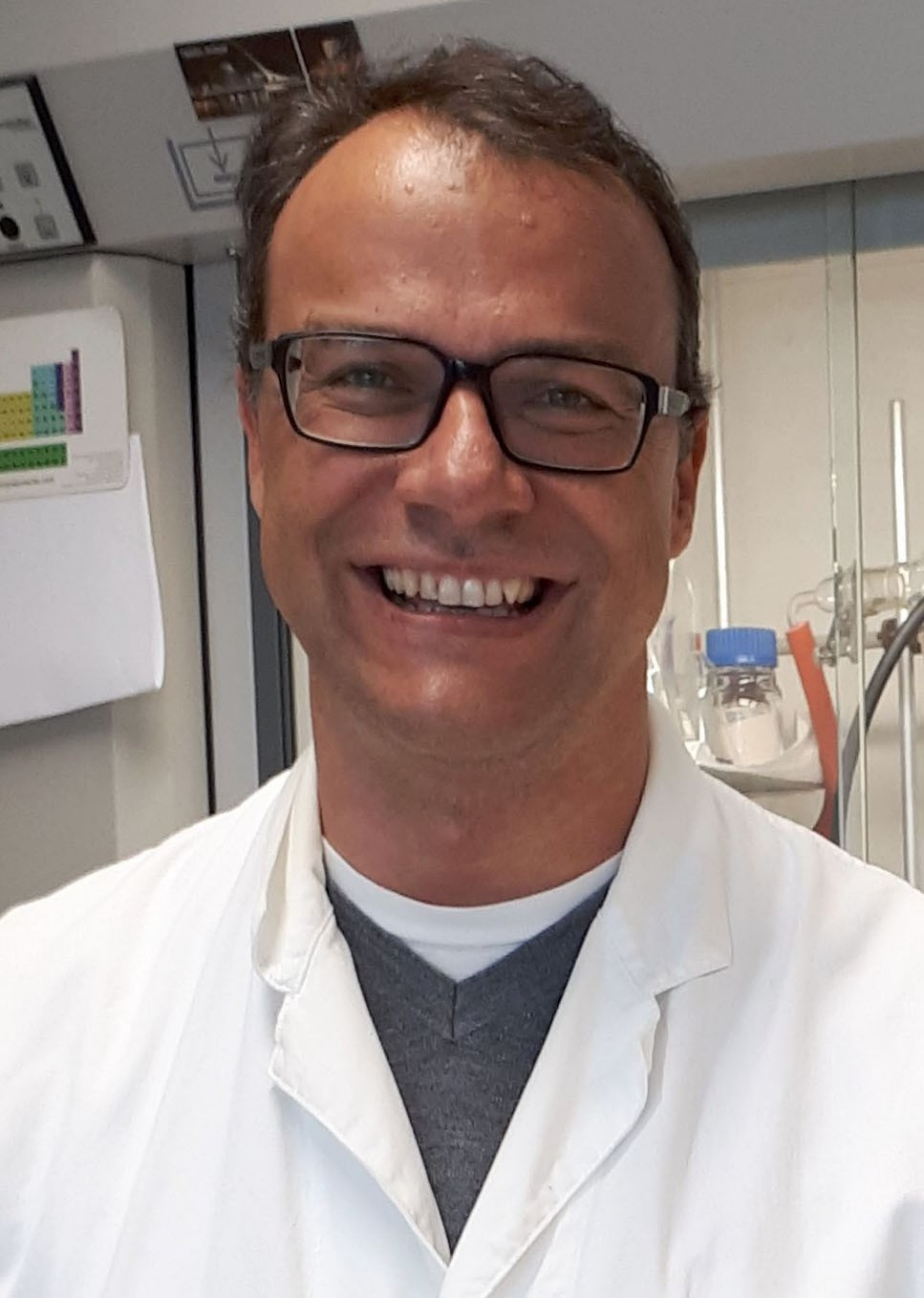# Synthesis of Hydrophilic Phosphorus Ligands and Their Application in Aqueous‐Phase Metal‐Catalyzed Reactions

**DOI:** 10.1002/adsc.202001278

**Published:** 2020-12-30

**Authors:** Thomas Schlatzer, Rolf Breinbauer

**Affiliations:** ^1^ Institute of Organic Chemistry Graz University of Technology Stremayrgasse 9 A-8010 Graz Austria phone

**Keywords:** Homogeneous catalysis, Ligand design, Phosphane ligands, Transition metals, Water chemistry

## Abstract

Transition metal‐catalyzed reactions in aqueous media are experiencing a constant increase in interest. In homogenous catalysis the use of water as a solvent offers advantages in cost, safety, the possibility of two‐phase catalysis and simplified separation strategies. In the life sciences, transition metal catalysis in aqueous systems enables the ligation or modification of biopolymers in buffer systems or even in their cellular environment. In biocatalysis, aqueous systems allow the simultaneous use of enzymes and transition metal catalysts in cascade reactions. The use of water‐soluble phosphine ligands still represents the most reliable and popular strategy for transferring metal catalysts into the aqueous phase. This review summarizes the recent advancements in this field since 2009 and describes current synthetic strategies for the preparation of hydrophilic phosphines and phosphites. In addition, recent applications of transition metal catalysis in aqueous solvents using these hydrophilic ligands are presented.

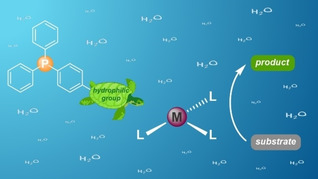

## 1 Introduction

Over the last decades homogeneous catalysis in aqueous environments has seen a continuous rise in interest. At the beginning the unique features of water as an inexpensive, intrinsically safe and environmentally benign solvent attracted the catalysis community. The iconic Ruhrchemie‐Rhône Poulenc process for the biphasic hydroformylation of propene using a Rh/TPPTS catalyst system on a >600.000 ton/a scale^[1]^ very early showed the tremendous potential of homogeneous catalysis in water. While these efforts continue, other lines of research have emerged, in which homogeneous catalysis is applied in the life sciences. It has been recognized that the distinct advantages of transition metal‐based homogeneous catalysis and biocatalysis can be combined when performed under the same solvent conditions.[^2^, ^3^, ^4^, ^5^] Transition metal complexes have also been used for the bioconjugation and modification of proteins.[^6^, ^7^, ^8^, ^9^, ^10^, ^11^] As most proteins denature in an organic solvent environment, it is very often necessary that water‐soluble metal complexes are used. The use of hydrophilic ligands represents the most straightforward approach to design such complexes.

In this article we aim to provide a comprehensive review about synthetic strategies towards novel hydrophilic phosphines and recent applications of hydrophilic ligands in aqueous media. It covers the time period from 2009, when the seminal review by Shaughnessy^[12]^ appeared, until present and should be understood to complement other reviews.[^13^, ^14^]

## 2 Current Strategies Towards Water‐Soluble Phosphorus Ligands

### 2.1 Sulfonate as Hydrophilic Group

The introduction of sulfonate groups still represents one of the most reliable and popular synthetic strategies for the preparation of water‐soluble phosphine ligands. This can be attributed to the combination of both synthetic accessibility and high hydrophilicity of sulfonate substituents. Although weakly Lewis basic sulfonate groups usually do not coordinate to late transition metals, it should be noted that especially vicinally sulfonated phosphines may act as κ^2^‐(*P*,*O*)‐chelating ligands.[^15^, ^16^, ^17^, ^18^] This section however will focus on ligands in which the sulfonate moiety functions as hydrophilic but not as coordinating group.

**Direct sulfonation**. The most straightforward approach is the direct electrophilic sulfonation of phosphine ligands by using H_2_SO_4_ or SO_3_/H_2_SO_4_ (oleum) as reported for the prototypic 3,3’,3’’‐phosphanetriyltris(benzenesulfonic acid) trisodium salt (*m*‐TPPTS)^[19]^ in the past. Under these strongly oxidizing and highly acidic conditions, the basic phosphine is protonated and thus protected from oxidation. However, the protonated phosphorus center deactivates adjacent aromatic rings, resulting in a *meta*‐directing effect as well as a requirement for harsh reaction conditions (high amount of oleum, high temperature, long reaction times) increasing the risk of phosphine oxidation. In order to minimize the amount of phosphine oxide as side product, several strategies have been developed. Through the addition of boric acid during sulfonation,^[20]^ an anhydride is formed which leads to the formation of a superacidic medium and removes SO_3_ (and its oxidative power). Careful pH‐adjustment during work‐up,^[21]^ or reduction of the resulting phosphine oxide (in the case of sulfonic acid esters)^[22]^ have also helped to address this issue.

**Scheme 1:**
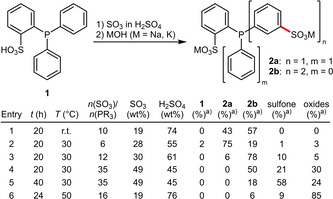
Preparation of non‐symmetrically sulfonated triphenylphosphines (^a)^ determined by ^31^P‐NMR spectroscopy).

Recently, in a systematic screening of the sulfonation conditions of (2‐diphenylphosphanyl)benzenesulfonic acid (*o*‐TPPMS, **1**) the key influence factors were identified as a) reaction temperature, b) concentration of SO_3_, c) concentration of H_2_SO_4_ and d) ratio *n*(SO_3_):*n*(phosphine).^[23]^ By careful adjustment of these parameters mono‐ or disulfonation leading to *rac*‐*o*,*m*‐TPPDS (**2 a**) and *o*,*m*,*m*‐TPPTS (**2 b**), respectively, were observed (Scheme 1). These non‐symmetrically sulfonated species combine the high negative charge density with κ^2^‐(*P*,*O*)‐coordinating properties. Prolonged reaction times and elevated temperatures however favoured the formation of the oxide *o*,*m*,*m*‐TPPTSO or a sulfone byproduct (upon dehydration of the *o*‐sulfonic acid and intramolecular cyclization with a phenyl substituent).

**Scheme 2:**
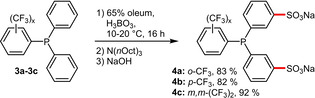
Sulfonation of electron‐deficient triarylphosphines.

Bayón et al. recently reported on the sulfonation of trifluoromethyl‐substituted triphenylphosphines **3 a**–**c** in presence of boric acid (Scheme 2). As expected, the electron‐withdrawing groups decrease the σ‐donating capability of ligands **4 a**–**c**, while increasing their π‐backbonding ability and robustness against oxidation.^[24]^

**Scheme 3:**
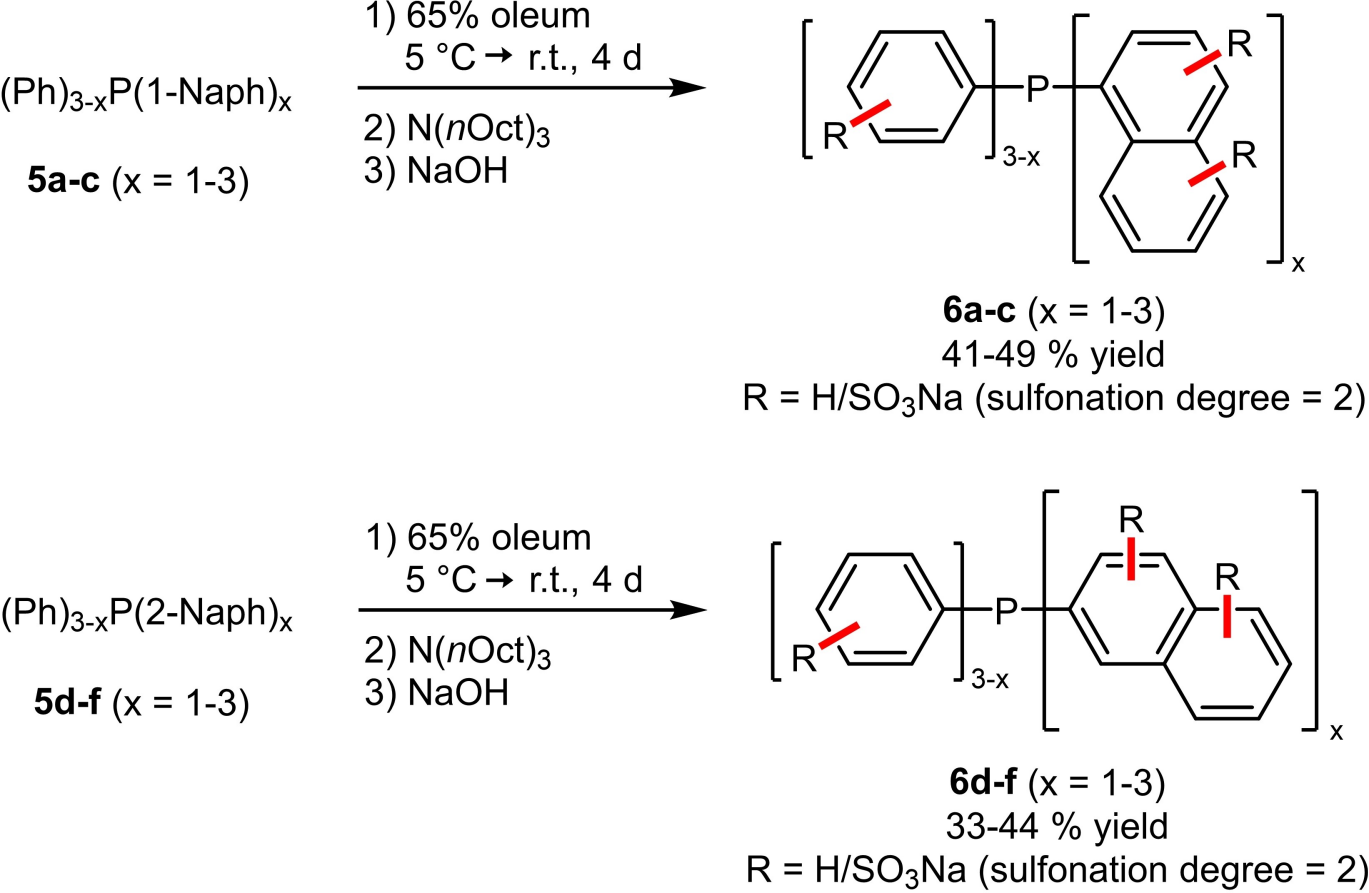
Sulfonation of naphthylphosphines.

In contrast to phenylphosphines, naphthylphosphines provide intrinsically more positions available for sulfonation but are also sterically more encumbered. Interestingly, even under drastic reaction conditions in a sulfuric acid/oleum mixture only a sulfonation degree close to 2 was observed for all six investigated phosphines (Scheme 3).^[25]^ This finding was attributed to the steric hindrance of the sulfonated naphthyl ring, which hampers a higher degree of sulfonation. However, it can be assumed that also a deactivating electronic influence of the electron‐withdrawing SO_3_H‐group has to be accounted.^[26]^

**Scheme 4:**
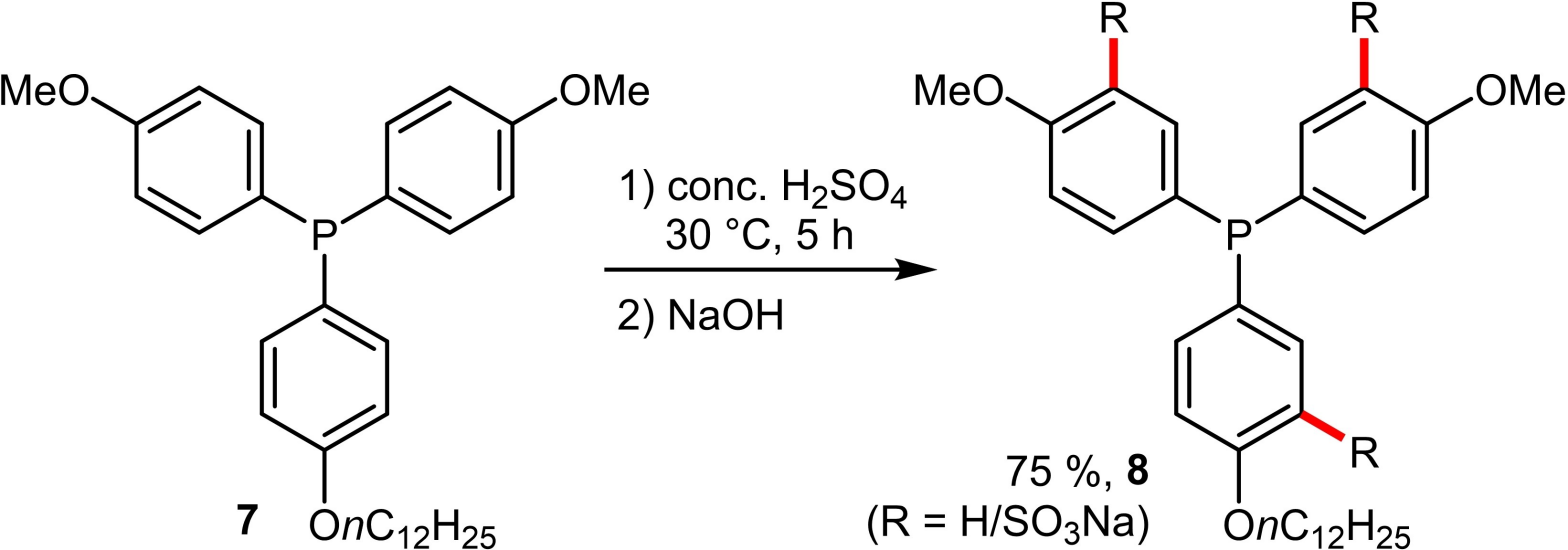
Sulfonation of an electron‐rich arylphosphine.

Arylphosphines that contain electron‐donating substituents readily undergo sulfonation in H_2_SO_4_ and do not require the use of strongly oxidizing oleum, as demonstrated in the preparation of the novel amphiphilic phosphine DMOPPS (**8**).^[27]^ However, a mixture of phosphines with a varying degree of sulfonation was obtained in this case (Scheme 4).

**Scheme 5:**
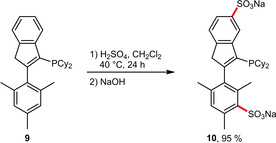
Disulfonation of a 2‐mesitylindenyl backbone.

Similarly, phosphino groups that are not directly attached to the aromatic moieties cannot deactivate these upon protonation. For this reason, such ligands can be easily sulfonated by treatment with conc. H_2_SO_4_. In a recent example the disulfonation of a 2‐mesitylindenyl‐containing phosphine **9**, that is structurally similar to XPhos, was reported.^[28]^ In this case not only the electron‐rich lower aromatic ring but also the upper indenyl system was functionalized affording the sulfonated ligand **10** in excellent yield (Scheme 5).

**Scheme 6:**
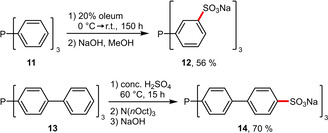
Comparison of the sulfonation of triphenylphosphine and tris(biphenyl)phosphine.

The concept of incorporating aromatic rings that are not in conjugation with the phosphine substituent is a useful tool in ligand design that significantly facilitates the sulfonation avoiding the use of oleum, which is reported to provoke the formation of phosphine oxides (up to 25% for TPPTS (**12**)).^[19],[29]^ Both the improved synthetic accessibility and the catalytic competitiveness were nicely demonstrated in a comparative study of sulfonated triphenylphosphine (**12**) and sulfonated tris(biphenyl)phosphine (**14**).^[30]^ It is noteworthy that in biphenyl substituents, always the more electron‐rich ring (i. e. which is not attached to the phosphorus atom) undergoes sulfonation (Scheme 6). As a consequence, the resulting ligand has a cone angle similar to the parent ligand triphenylphosphine, while *meta*‐sulfonated TPPTS is sterically more congested resulting in a larger cone angle.^[31]^

**Scheme 7:**
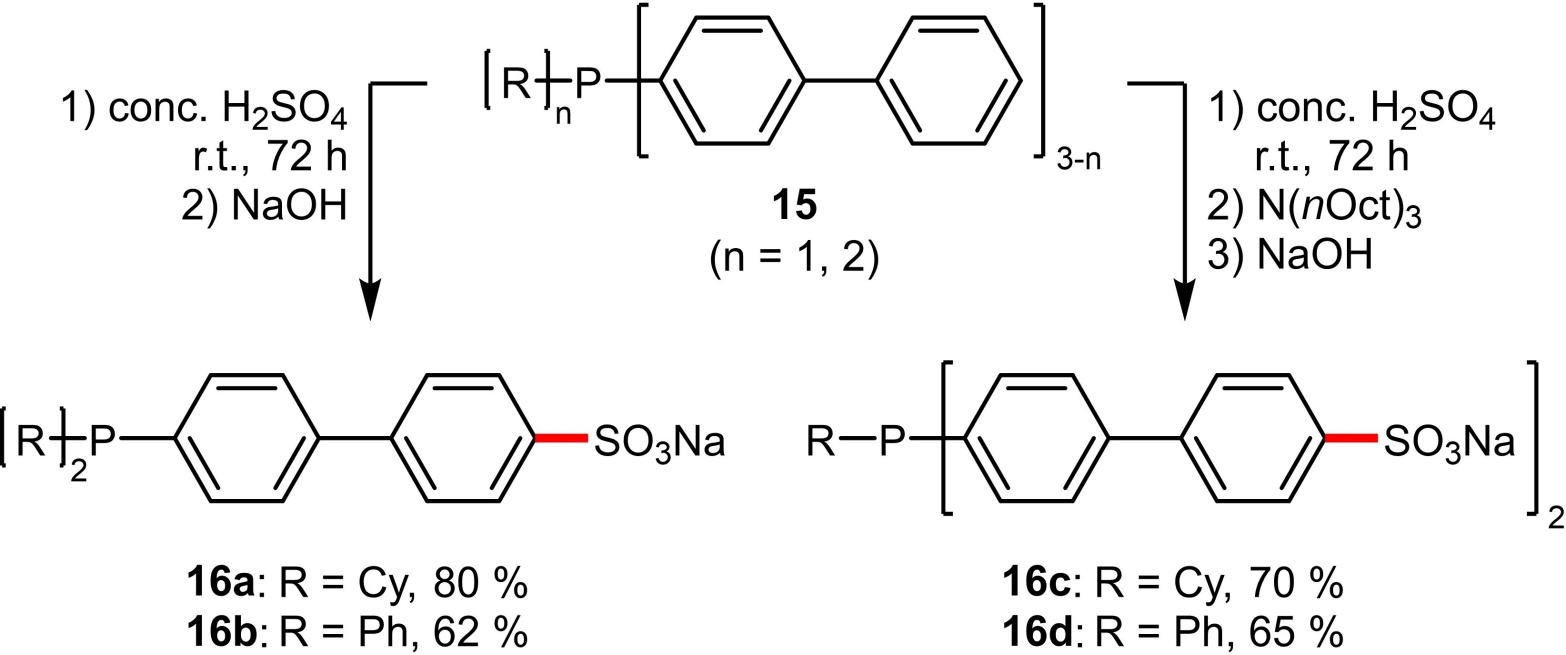
Sulfonation of biphenyl‐containing phosphines.

The same rationale was applied in the synthesis of the amphiphilic phosphines **16 a**–**d** (Scheme 7). In all cases exclusively the remote phenyl moieties underwent *para*‐sulfonation, while aryl rings directly attached to the phosphorus atom remained pristine. The functionalized ligands were isolated in high yields and exhibited good to excellent water solubilities depending on the number of sulfonate groups introduced (**16 a**: 50 g L^−1^, **16 b**: 280 g L^−1^, **16 c**: 520 g L^−1^, **16 d**: 850 g L^−1^).^[32]^

**Scheme 8:**
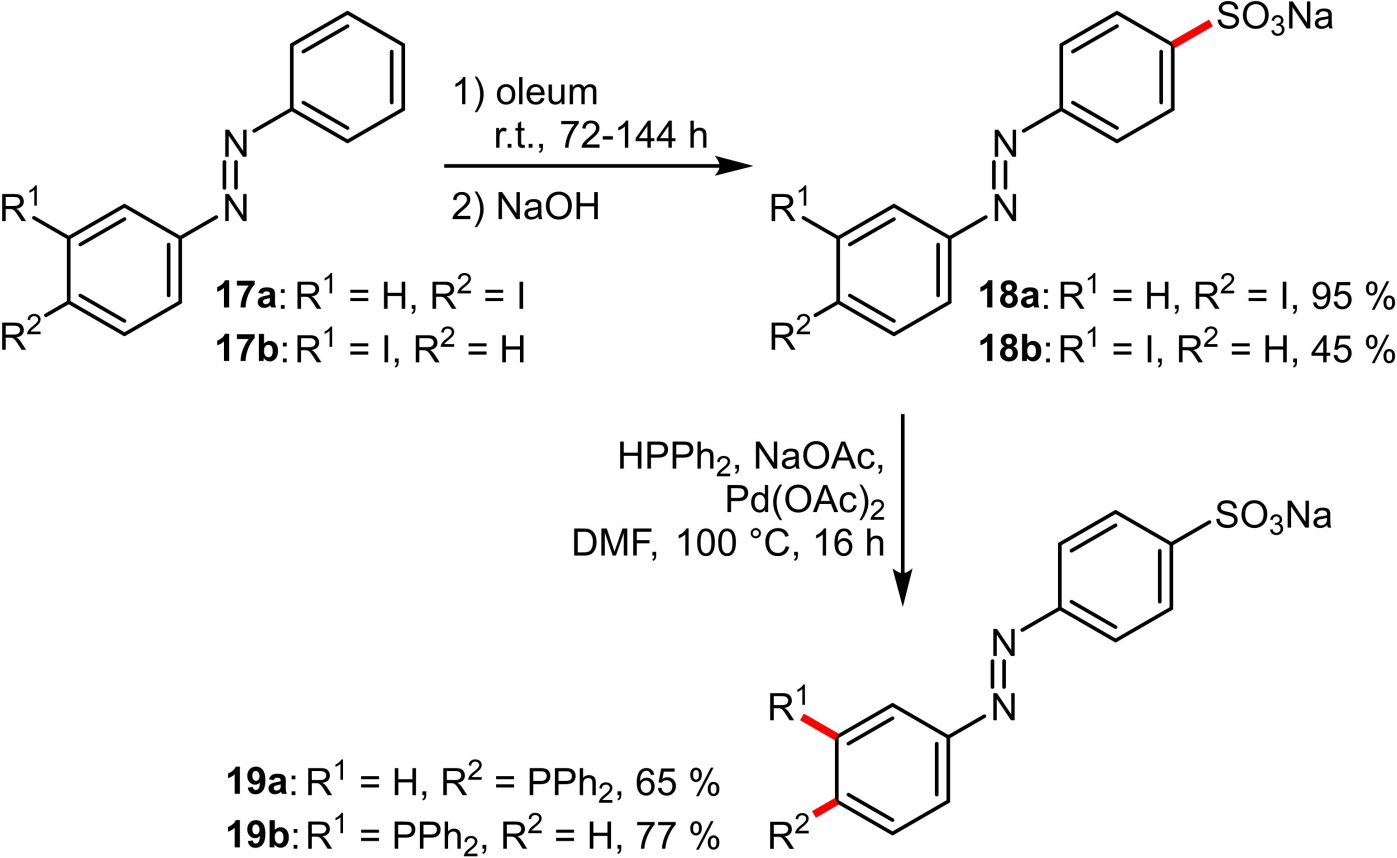
Preparation of diazo‐containing water‐soluble phosphines.

For some cases it might be advantageous to sulfonate the aromatic scaffold prior to the introduction of the phosphine. This strategy was pursued in the preparation of water‐soluble, UV‐switchable ligands (Scheme 8). To this end, iodinated azobenzenes **17 a** and **17 b** were sulfonated first (the low yield in the case of **18 b** was due to unselective sulfonation). Afterwards the phosphine substituent was introduced at the predefined, iodinated position by a Pd‐catalyzed reaction with diphenylphosphine. Interestingly, the ligands **19 a** and **19 b** were shown to exhibit a lower basicity compared to the structurally similar **16 b**, reflecting the electron‐withdrawing effect of the diazo group.^[33]^ It has to be noted that the diazo group, despite its electron‐withdrawing nature,^[26]^ is an *ortho*/*para*‐directing substituent.

Usually sulfonate groups which are only weakly Lewis basic do not bind strongly to the metal center of complexes. However, if the sulfonate and phosphine substituents are in proximity, κ^2^‐(*P*,*O*)‐chelating coordination to transition metals is feasible.[^15^, ^16^, ^17^, ^18^]

**Scheme 9:**
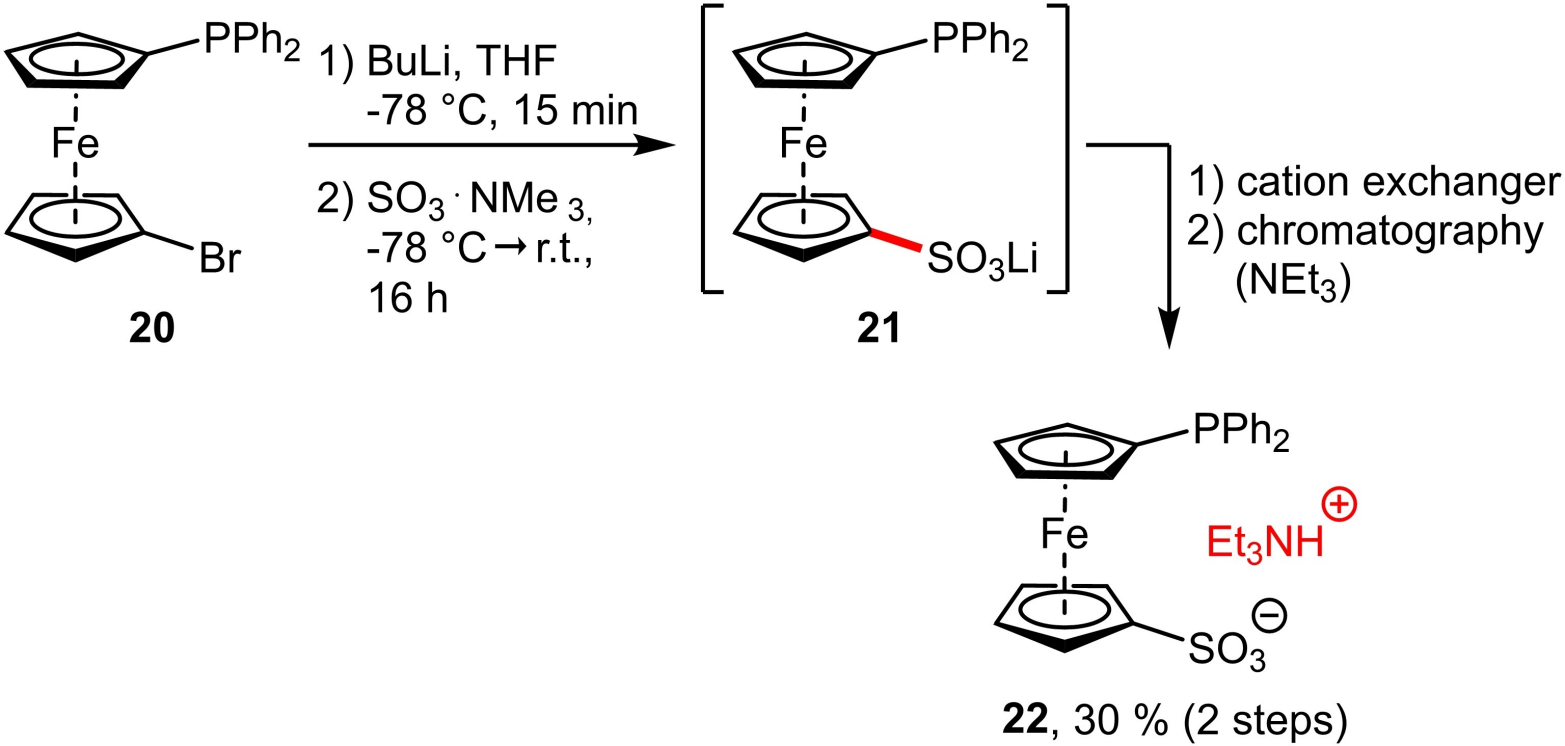
Synthesis of sulfonated phosphinoferrocene **22**.

A recent example for the development of a hybrid ligand featuring an ambivalent sulfonate group, which exhibits both hydrophilic and Lewis donating properties, was reported by Štěpnička et al.^[34]^ A strategically positioned bromine substituent enabled lithium‐halogen‐exchange at this position. The sulfonate group was installed via electrophilic quench with SO_3_⋅NMe_3_ affording the water‐soluble ligand **22** (Scheme 9). Depending on the palladium precursor, **22** can either chelate Pd in a κ^2^‐(*P*,*O*)‐coordinating mode or function as a monodentate ligand with a free sulfonate group. In the latter case the resulting complex is expected to be more hydrophilic, indicating that the tendency of the sulfonate to participate in coordination can influence its water‐solubilizing capability.

**Incorporation of Sulfonated Precursors**. As already mentioned in the introduction to this chapter, the harsh reaction conditions usually associated with direct sulfonation are not compatible with all structural features of common phosphine ligands. Examples for oleum‐sensitive groups on alkyldiphenylphosphines are long alkyl chains (>C_6_) or alkenyl substituents.^[35]^ While alkyl chains longer than hexyl are cleaved,^[36]^ the double bond of an alkenyl moiety would be protonated and thus susceptible to side reactions.^[35]^

**Scheme 10:**
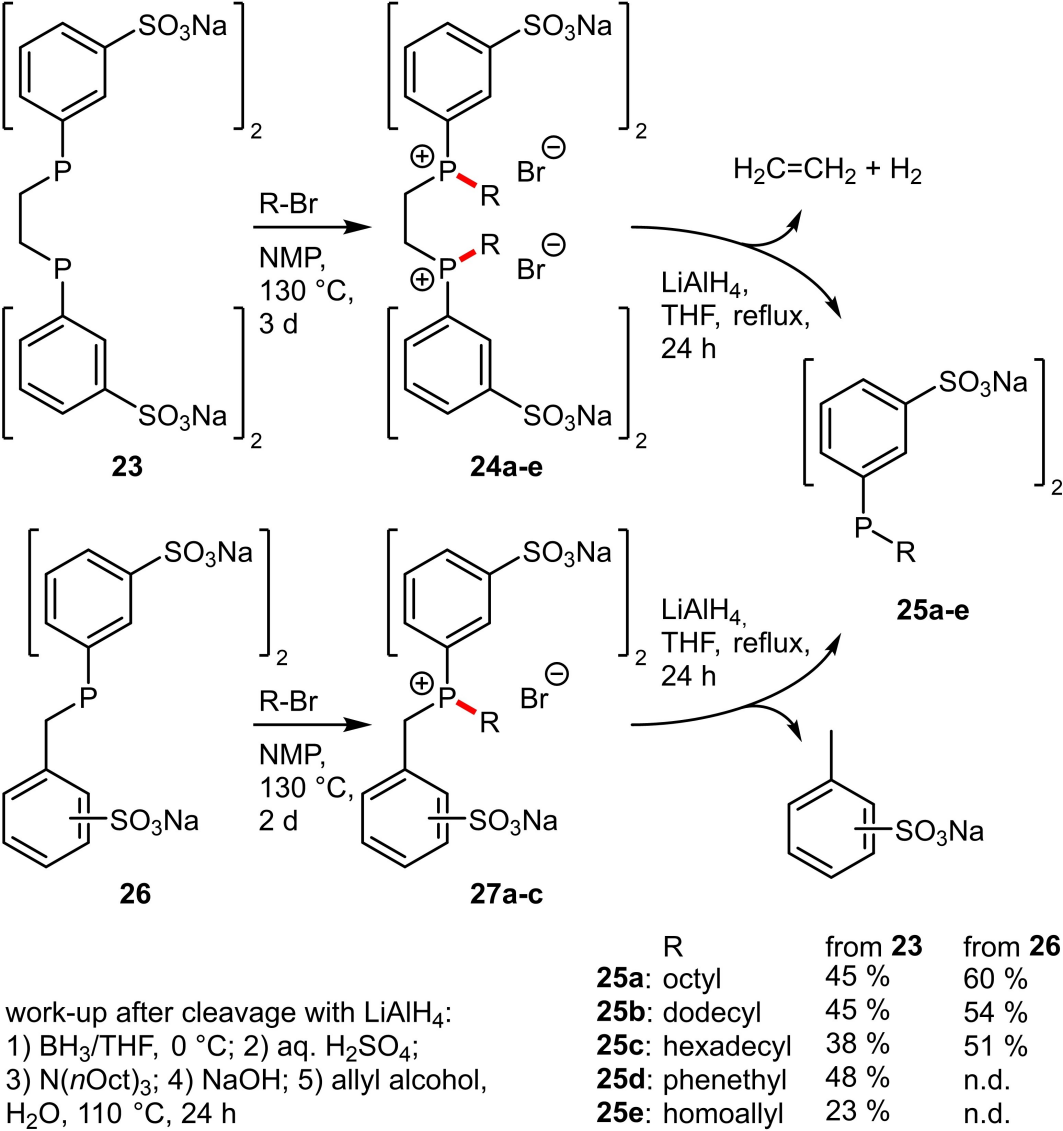
Synthesis of disulfonated alkyldiphenylphosphines.

For this purpose, the group of Monflier reported two elegant strategies that introduce these oleum‐sensitive groups subsequently to the sulfonation reaction.^[35],[37]^ Starting from sulfonated precursors DPPETS (**23**) or BDPPTS (**26**) the oleum‐sensitive group was installed by alkylation of the phosphine. The resulting phosphonium intermediates were then reductively cleaved by LiAlH_4_ to liberate the disulfonated alkyldiphenylphosphines **25 a**–**e** (Scheme 10). Given the better accessibility of BDPPTS (**26**) and the consequential higher overall yield, the second pathway seems superior.^[37]^

**Scheme 11:**
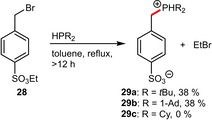
Benzylic substitution and concomitant sulfonate ester cleavage.

Another appealing alternative to the tedious direct sulfonation of benzylic phosphines was recently described by Shaughnessy et al.^[38]^ The sulfonate group was envisioned to be installed prior to the alkylation of the phosphorus center and protected as ethyl ester. The sulfonated benzylic substrate **28** was then treated with secondary alkylphosphines. It is noteworthy that the resulting bromide instantly liberates the sulfonated ligand from the ethyl protecting group yielding the desired functionalized ligands **29 a** and **29 b** in a single step (Scheme 11). The corresponding dicyclohexyl derivative **29 c** however could not be obtained following this protocol.

**Scheme 12:**
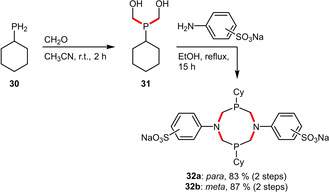
Preparation of sulfonated diphosphadiazacyclooctane ligands.

Depending on the structure of the ligand, the sulfonate group might also be preinstalled on a building block and thus incorporated during ligand assembly. This has been demonstrated by Monflier et al. for the synthesis of bidentate diphosphadiazacyclooctanes. The reasonably water‐soluble (6.7 g L^−1^) phosphines **32 a** and **32 b** were prepared in two steps and high yields (Scheme 12).^[39]^

**Scheme 13:**
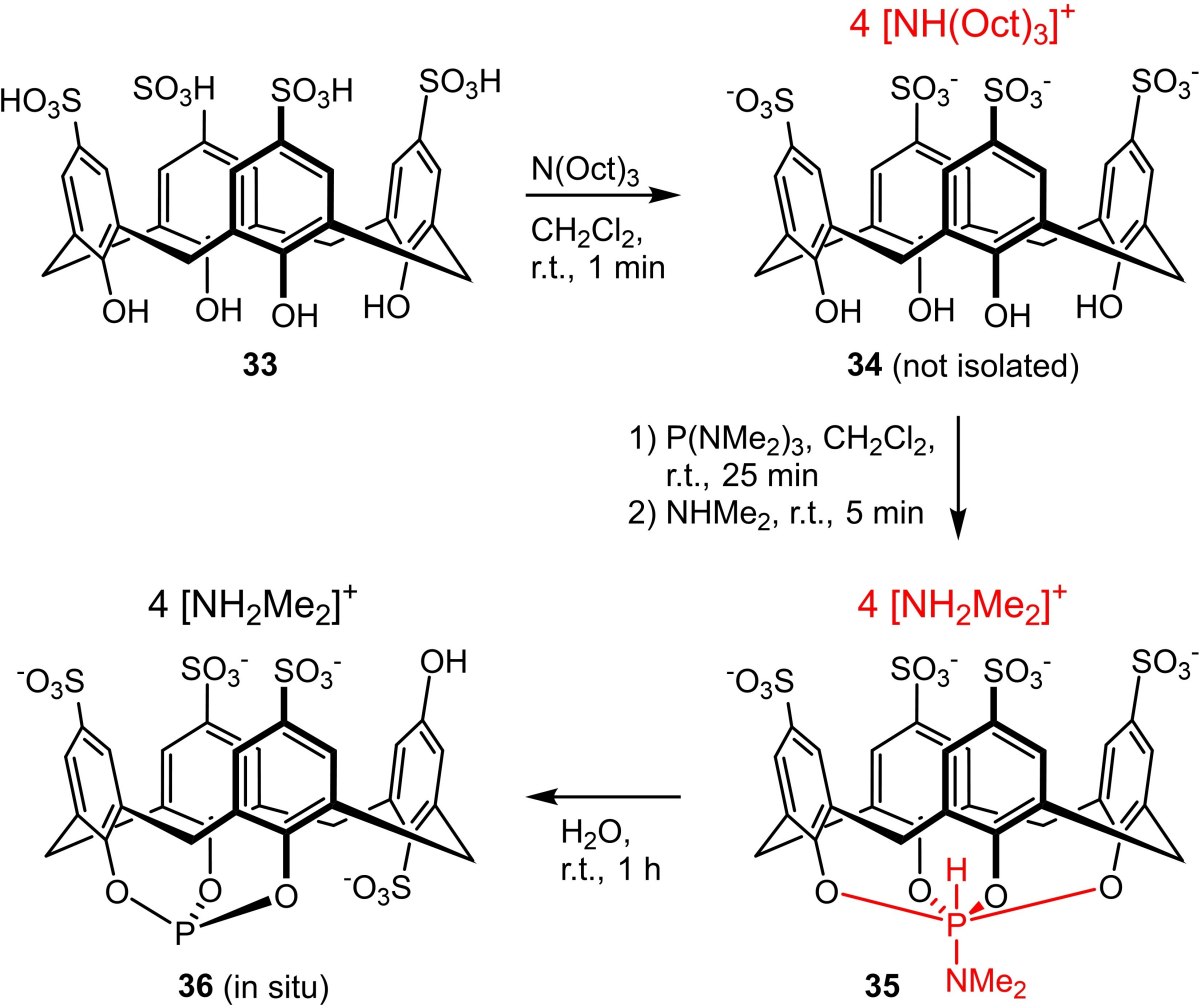
Synthesis of a phosphite ligand derived from sulfonated calix[4]arene.

Pringle et al. reported the novel water‐soluble phosphite ligand **36** based on a sulfonated calix[4]arene scaffold. Starting from the sulfonated precursor **33**, the six‐coordinate phosphorus species **35** was obtained in one‐pot (Scheme 13). Upon treatment with water, the rather sensitive phosphite **36** is generated, which decomposes over 24 h. Remarkably, the corresponding rhodium(I) complex is much more resistant to hydrolysis exhibiting a half‐life in water of 4 months.^[40]^

**Linker‐based Strategies**. A fundamentally different strategy towards water‐soluble phosphines is the attachment of hydrophilic tags onto reactive handles of prefunctionalized ligands. Such stable sulfonated building blocks can substantially facilitate the overall ligand assembly. The group of Štěpnička has recently published several applications of short chain ω‐aminosulfonic acids H_2_N(CH_2_)_n_SO_3_H (n=1–3) that can be linked to carboxylated ligands via an amide bond and will be covered in this section.[^41^, ^42^, ^43^] For a comprehensive overview of phosphino‐carboxamides exceeding hydrophilic applications the reader is directed to another review.^[44]^

**Scheme 14:**
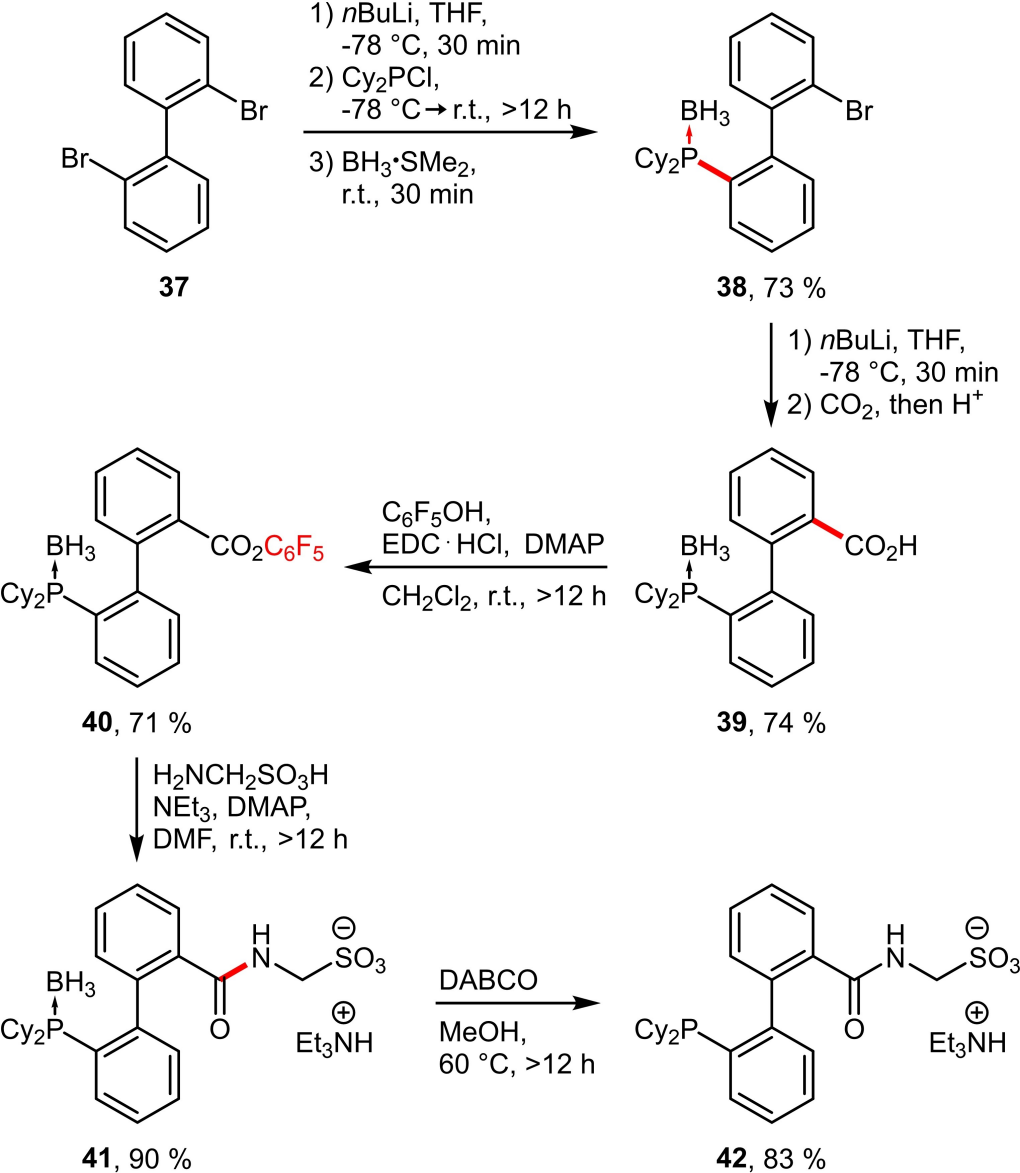
Synthesis of amidophosphine **42**.

A representative synthesis following this approach is given in Scheme 14.^[41]^ Both the phosphino as well as the carboxy group are successively installed via lithiation and electrophilic quench with ClPR_2_ and CO_2_, respectively. Subsequently, the carboxylic acid **39** can be transformed into the active pentafluorophenyl ester **40**, which readily undergoes amidation with aminomethylsulfonic acid, affording the functionalized ligand in its protected form.

**Scheme 15:**
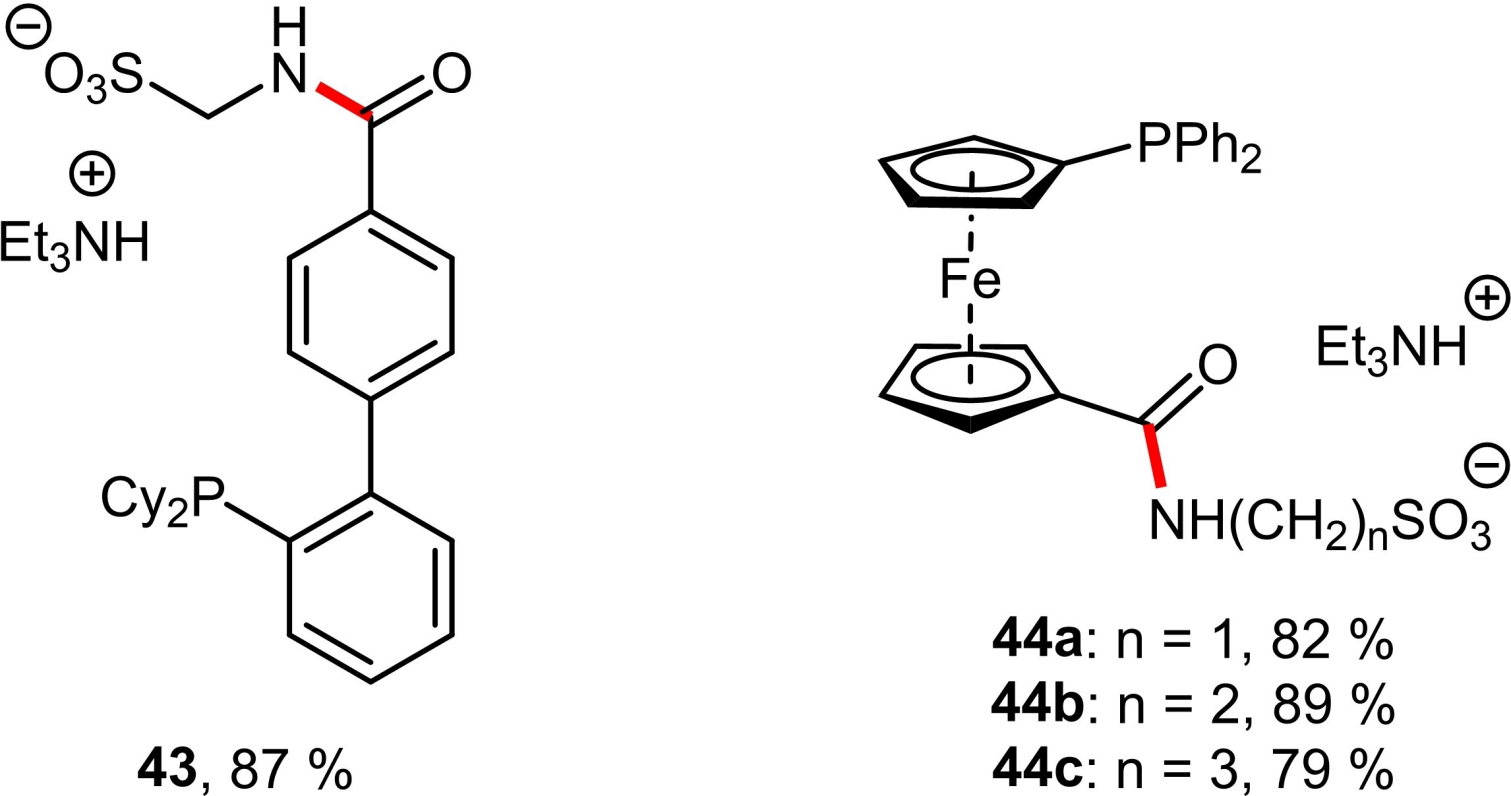
Additional amidophosphines prepared analogously (yields refer to the amidation step).

The sterically less encumbered isomer **43** (Scheme 15) was prepared in similar manner.^[42]^ Moreover, this concept has been also successfully extended to structurally different phosphinoferrocenes **44 a**–**c** (Scheme 15) using ω‐aminosulfonic acids H_2_N(CH_2_)_n_SO_3_H with varying chain lengths (n=1–3).^[43]^

**Scheme 16:**
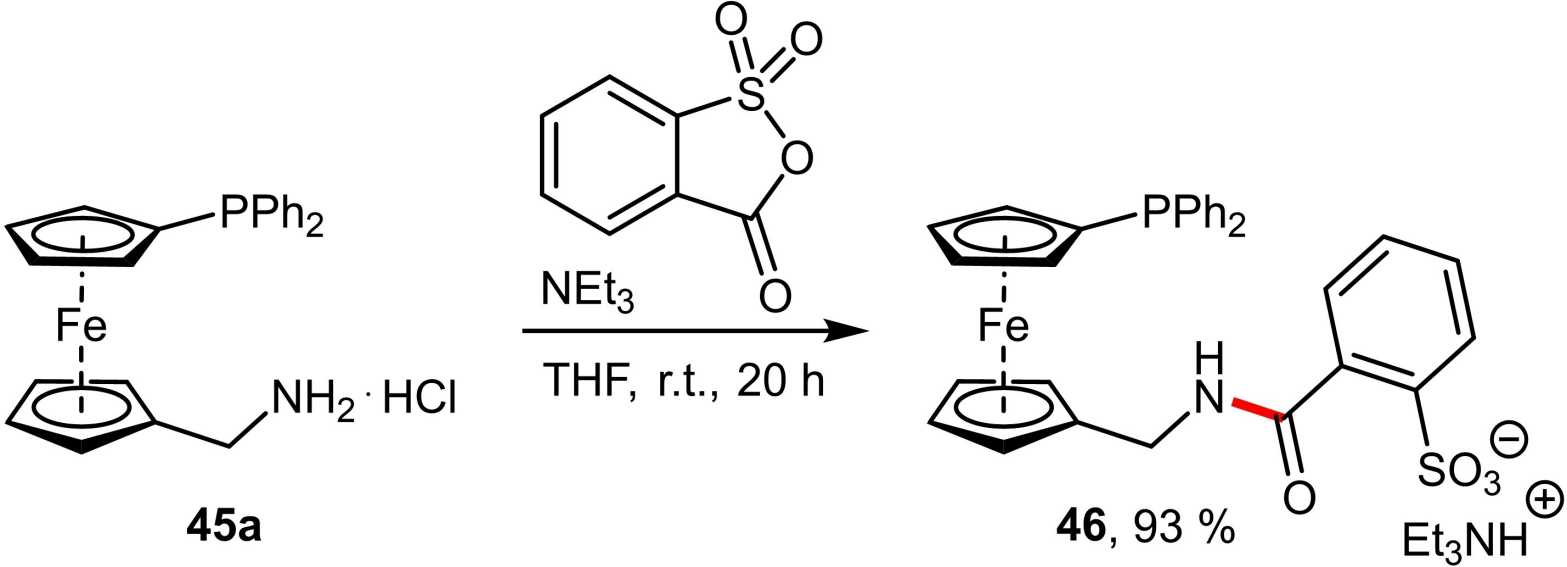
Preparation of a hydrophilically tagged phosphinoferrocene with an inverted amide linkage.

In an effort to expand the family of hydrophilically tagged phosphinoferrocenes, the orientation of the amide linkage was formally inverted starting from the methylamine functionalized phosphine **45 a**. The reagent 2‐sulfobenzoic anhydride is ideally suited for this purpose since it exhibits the required electrophilicity and liberates the hydrophilic sulfonate group upon reaction (Scheme 16). Although the resulting ligand **46** is hygroscopic, it is stable in air. In contrast, the reaction of **45 a** with phthalic anhydride was unsuccessful since the resulting amide is unstable and undergoes hydrolysis.^[45]^

### 2.2 Other Anionic Hydrophilic Groups

**Scheme 17:**
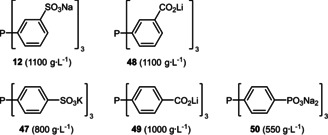
Comparison of the hydrophilic properties of sulfonate, carboxylate and phosphonate groups in triarylphosphines (solubilities in water are given in parentheses).

Besides the most popular sulfonate group, in principle any other anionic and thus polar substituent can be considered as hydrophilic entity in the design of water‐soluble ligands. Typical examples encompass carboxylic or phosphonic acids for this purpose. The most important difference however is the lack of any direct access (e. g. by electrophilic aromatic substitution) starting from the respective parent phosphine. This might account for the lower quantity of such ligands even though the water‐solubilizing ability of carboxylates and phosphonates is comparable to sulfonate groups (Scheme 17).[^46^, ^47^, ^48^, ^49^]

**Scheme 18:**
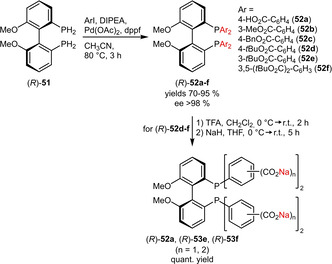
Synthesis of atropisomeric phosphines containing carboxylate groups.

**Carboxylates**. A related contribution reported a Pd‐catalyzed P−C coupling reaction to prepare atropisomeric MeOBIPHEP ligands from primary phosphines.^[50]^ This racemization‐free protocol enabled the preparation of several phosphine ligands containing carboxylic esters (**52 b**–**f**) and even a free carboxylic acid (**52 a**) in good to excellent yields (Scheme 18). In a follow‐up article the potential of this methodology was confirmed since the *t*Bu esters (**52 d**–**f**) could be efficiently deprotected by trifluoroacetic acid and converted to the sodium salts, which were readily water‐soluble (**52 a**: 175 g L^−1^, **53 e**: 130 g L^−1^, **53 f**: 385 g L^−1^).^[51]^ Although these studies focused on carboxylated MeOBIPHEP derivatives, the underlying concept might be applicable to other ligand systems such as the closely related BINAP for example.

**Scheme 19:**
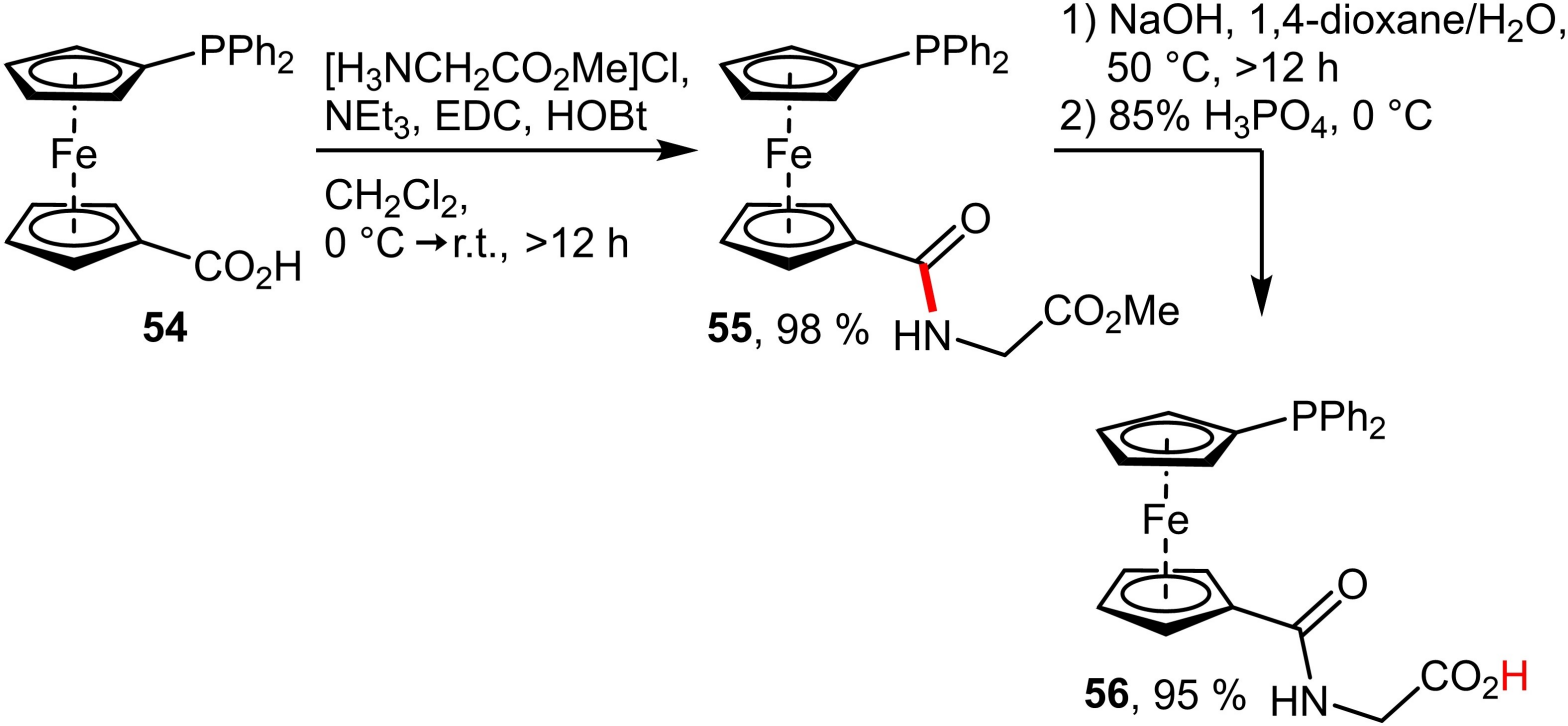
Preparation of a glycine‐tagged phosphinoferrocene.

Similar to ω‐aminosulfonic acids also classical α‐amino acids such as glycine can be attached to carboxylated phosphine ligands (Scheme 19).^[52]^ However, following this approach, the resulting amide linkage represents the only additional polar moiety.

**Scheme 20:**
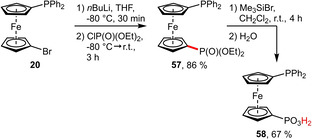
Synthesis of 1’‐(diphenylphosphino)ferrocene‐1‐phosphonic acid (**58**).

**Phosphonates**. Very recently, the phosphonic acid functionalized diphenylphosphinoferrocene ligand **58** was synthesized, completing the series of diphenylphosphinoferrocene ligands featuring hydrophilic substituents (sulfonate, carboxylate, phosphonate). Since the corresponding phosphonate ester **57** ^[53]^ has already been reported, the free phosphonic acid **58** could be obtained by Me_3_SiBr‐mediated hydrolysis in good yield (Scheme 20).^[54]^ Although the free acid was found to be unstable even at 4 °C, it can be employed for the preparation of Pd‐complexes. Depending on the protonation state, this ligand can either act as a monodentate *P*‐donor (in the fully protonated form) or as *P*,*O*‐chelating ligand upon deprotonation to the monoanion of **58**.

### 2.3 Ammonium as Hydrophilic Group

Analogously to negatively charged substituents (sulfonate, carboxylate, phosphonate), positively charged groups are also very frequently encountered. Functional groups containing basic nitrogen atoms (e. g. aliphatic amines, guanidines, *N*‐heterocycles), which upon protonation or alkylation form hydrophilic ammonium species, were most frequently encountered in the last decade. While alkylation provides the ligand with a permanent charge, protonated species can be considered as amphiphiles whose solubility depend on the pH of the solvent. However, this might be incompatible with the intended catalytic reaction conditions.

**Scheme 21:**
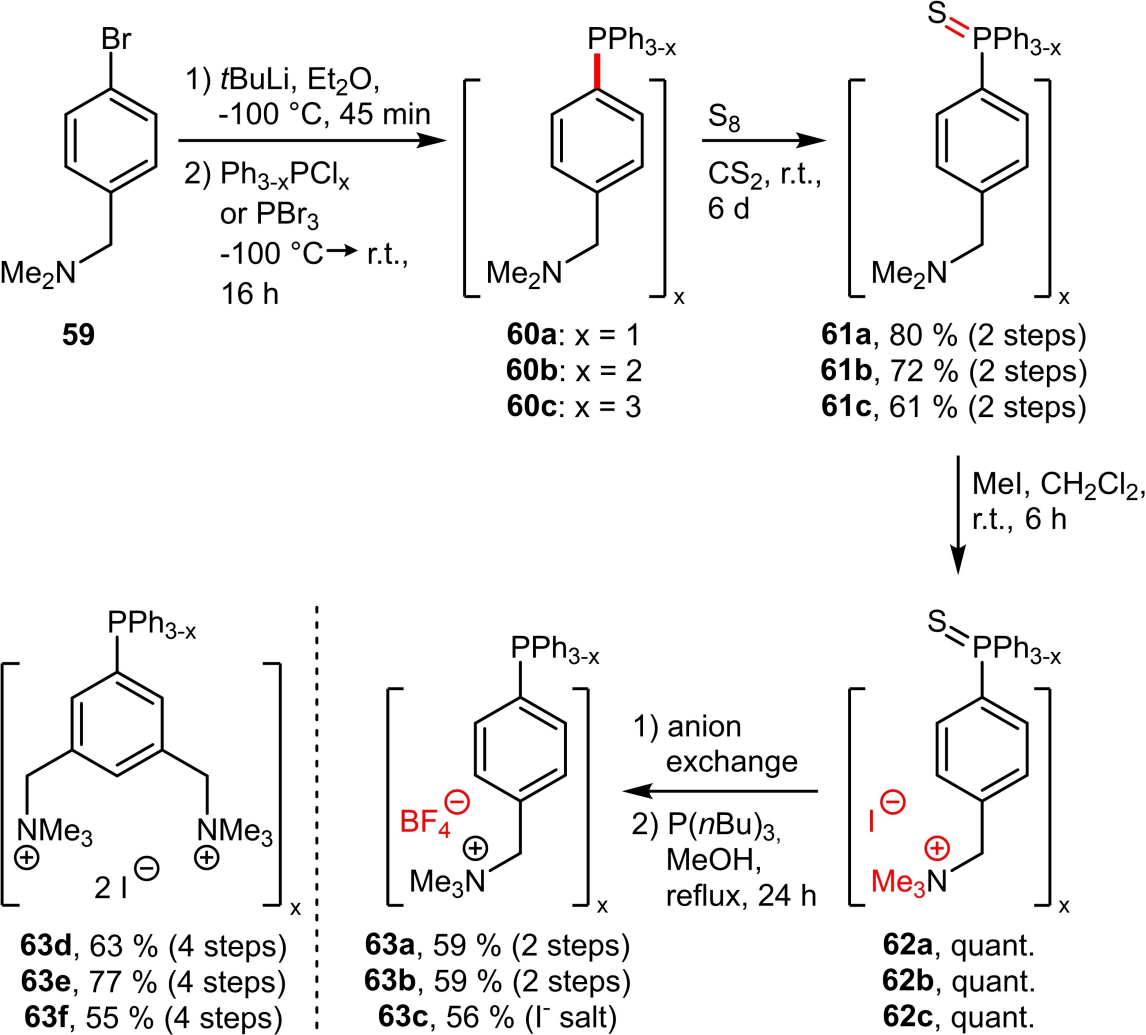
Preparation of ammoniomethyl‐substituted triaryl phosphines.

**Aliphatic Amines**. The groups of Klein Gebbink and Laurenczy have recently expanded the series of ammoniomethyl‐substituted triaryl phosphines[^55^, ^56^] based on the initial literature precedent (**63 f**) from 2003.^[57]^ The common synthetic strategy (Scheme 21) employs (dimethylamino)methyl‐substituted bromobenzenes for the preparation of the corresponding triaryl phosphines. In order to convert the tertiary amines into the hydrophilic ammonium groups, the phosphorus atom had to be protected temporarily as phosphine sulfide to enable selective *N*‐alkylation. The number of ammoniomethyl groups does not only influence steric and hydrophilic properties but also the basicity of the phosphine, which was found to be similar to *m*TPPTS for **63 c**.^[56]^

**Scheme 22:**
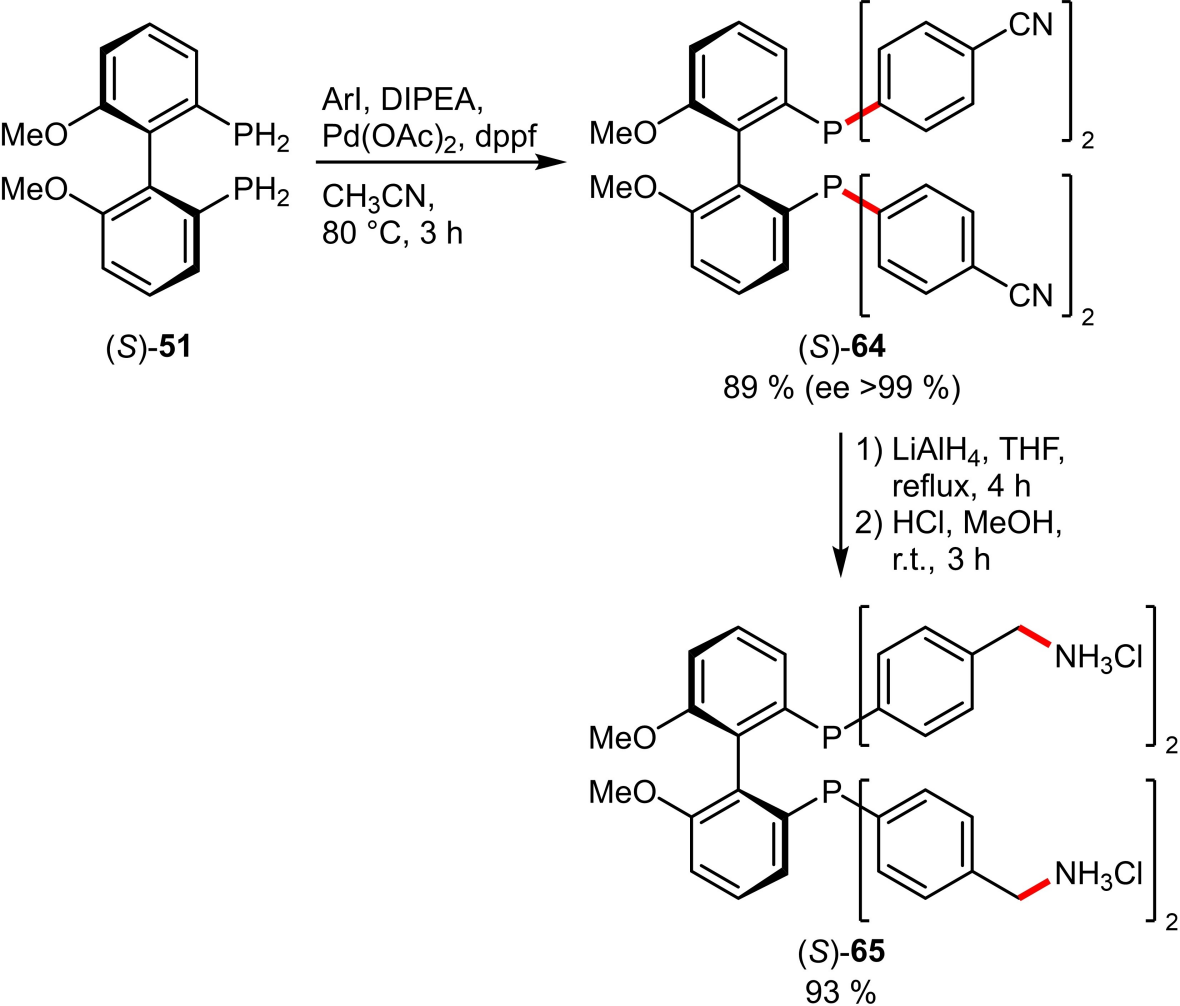
MeOBIPHEP derivative containing ammoniomethyl groups.

Aliphatic amines were also incorporated into MeOBIPHEP ligands. To this end, a cyano‐substituted aryl iodide was subjected to a Pd‐catalyzed P−C coupling as already introduced in Scheme 18. The cyano functionalities were subsequently reduced to methylamino groups followed by protonation. This high yielding protocol afforded the water‐soluble (110 g L^−1^) derivative **65** (Scheme 22).^[51]^

**Scheme 23:**
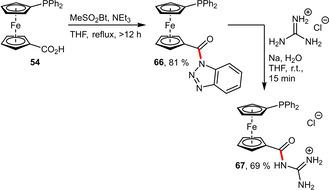
Installation of an acylguanidinium substituent on a phosphinylferrocene.

**Guanidinium Groups**. Recently, the guanidine moiety as hydrophilic entity was revisited by the group of Štěpnička for the preparation of phosphinylferrocene ligands.[^58^, ^59^, ^60^] Taking advantage of a carboxyl group, which can be transformed into its acylbenzotriazole derivative, an acylguanidinium moiety could be efficiently installed (Scheme 23).^[58]^

**Scheme 24:**
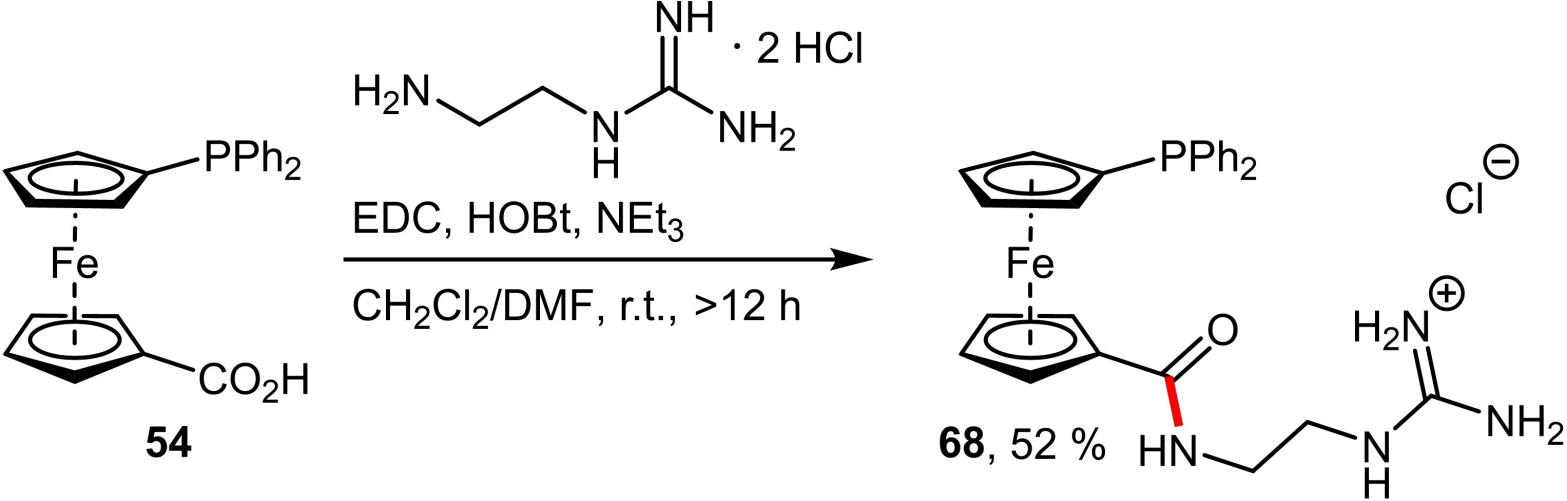
Synthesis of the corresponding alkylguanidinium ligand **68**.

The corresponding alkylguanidinium ligand **68** incorporating an aliphatic spacer was synthesized by a standard amide coupling reaction (Scheme 24).^[58]^ Both ligands (**67** and **68**) were air stable and although not particularly water‐soluble they could be used for Pd‐catalyzed cross‐couplings in biphasic reaction media.

**Scheme 25:**
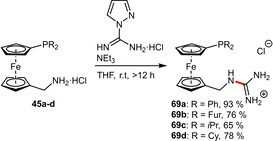
Direct guanylation of aminomethyl‐substituted phosphinoferrocenes.

Starting from the related aminomethyl‐substituted diphenylphosphinoferrocene **45 a**, direct guanylation using 1*H*‐pyrazole‐1‐carboximidamide hydrochloride readily afforded the desired ligand **69 a** in excellent yield.^[59]^ The same protocol was employed for the preparation of similar ligands (**69 b**–**d**) differing in the alkyl/aryl groups at the phosphorus atom (Scheme 25).^[60]^

**Scheme 26:**
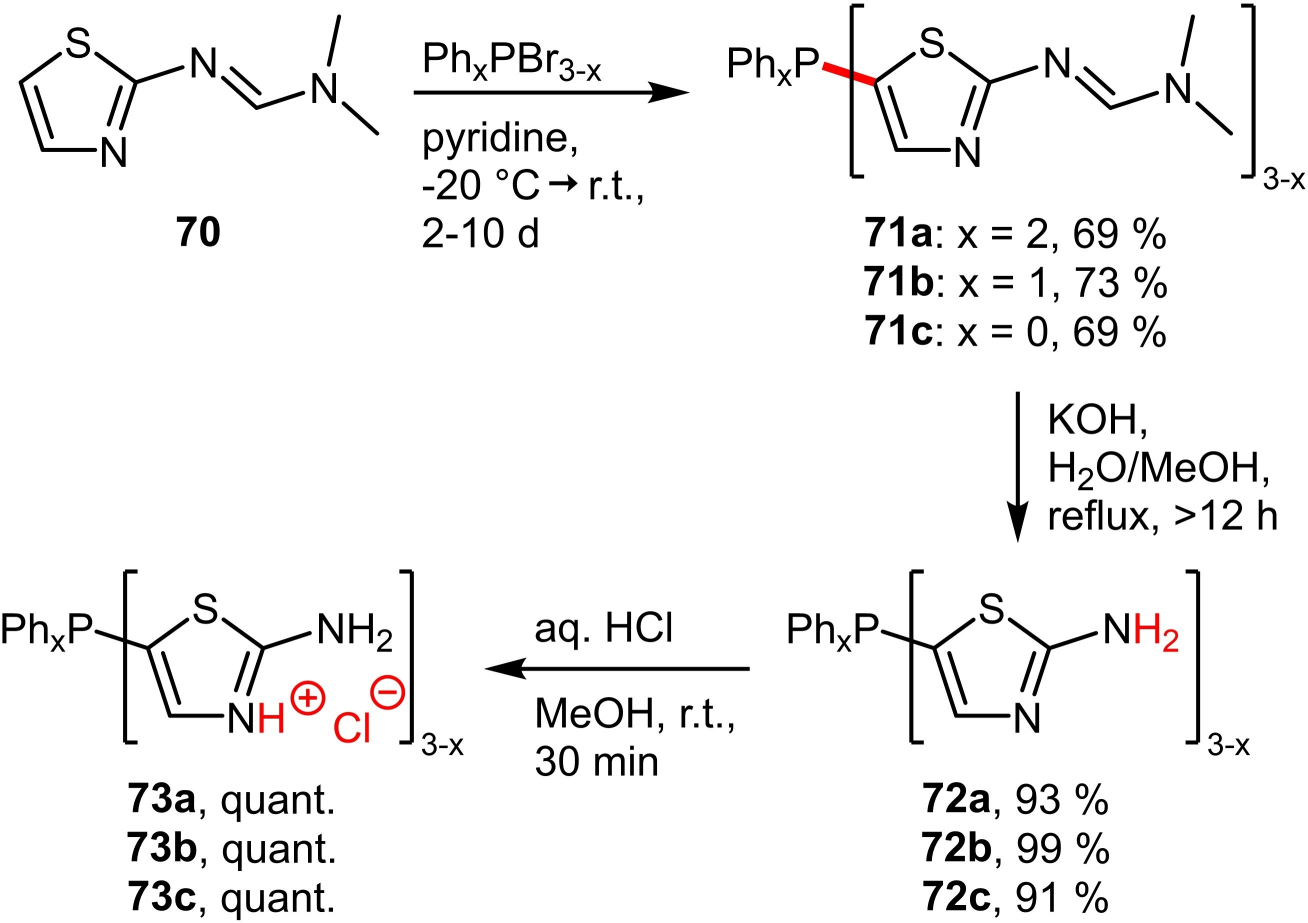
Synthesis of thiazolyl‐phosphines.

***N*** **‐Heterocycles**. Cadierno and coworkers identified a group of 5‐(2‐aminothiazolyl)‐phosphines (**72 a**–**c**) from the literature^[61],[62]^ and explored their potential as precursors for hydrophilic ligands. To this end, the 2‐aminothiazolyl groups were selectively protonated at the more basic imine nitrogen atoms affording modestly (**73 a**: 6 g L^−1^) and highly (**73 b**: 250 g L^−1^, **73 c**: 275 g L^−1^) water‐soluble phosphines (Scheme 26).^[63]^

**Scheme 27:**
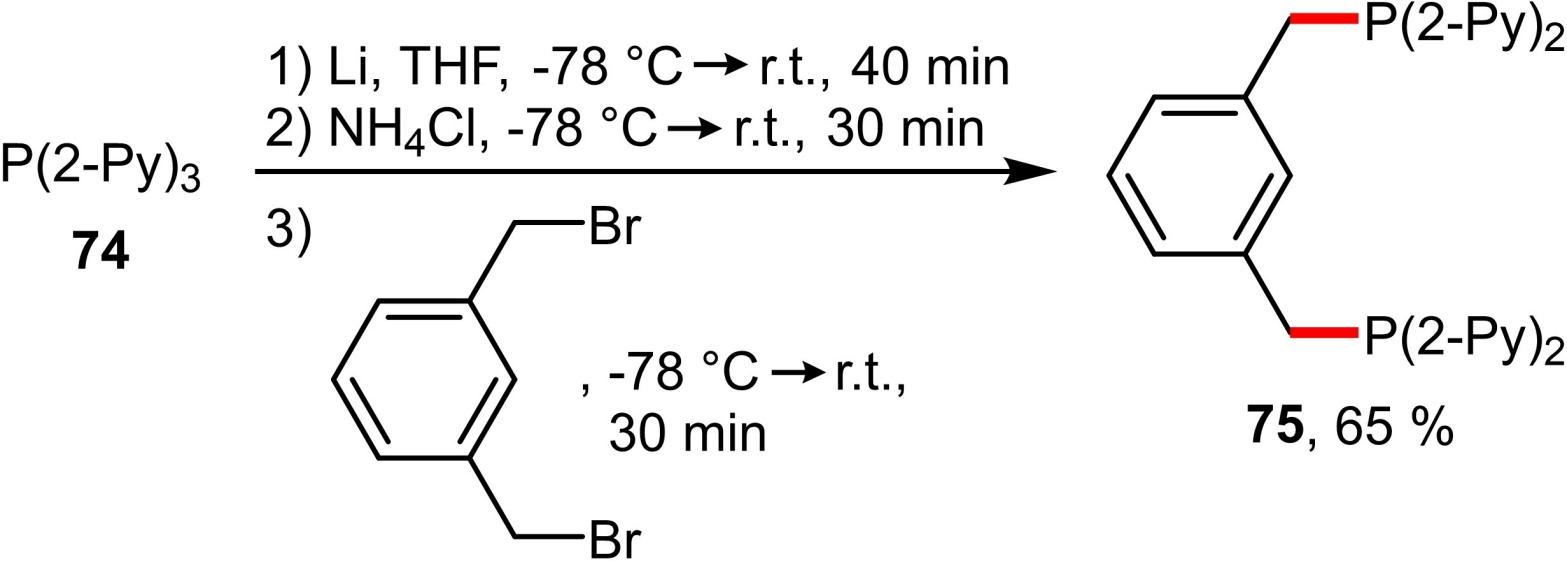
Incorporation of 2‐pyridyl moieties, which can be protonated to enhance their hydrophilicity.

Analogously, the water‐solubility of a pyridine‐containing pincer ligand **75** could be significantly increased by simple protonation with HCl. The phosphine **75** was readily obtained starting from dibromo‐*m*‐xylene and in situ prepared lithium bis(2‐pyridyl)phosphide (Scheme 27). Due to the electron‐withdrawing properties of the 2‐pyridyl substituents **75** was found to be slightly less basic than PPh_3_.^[64]^

**Scheme 28:**
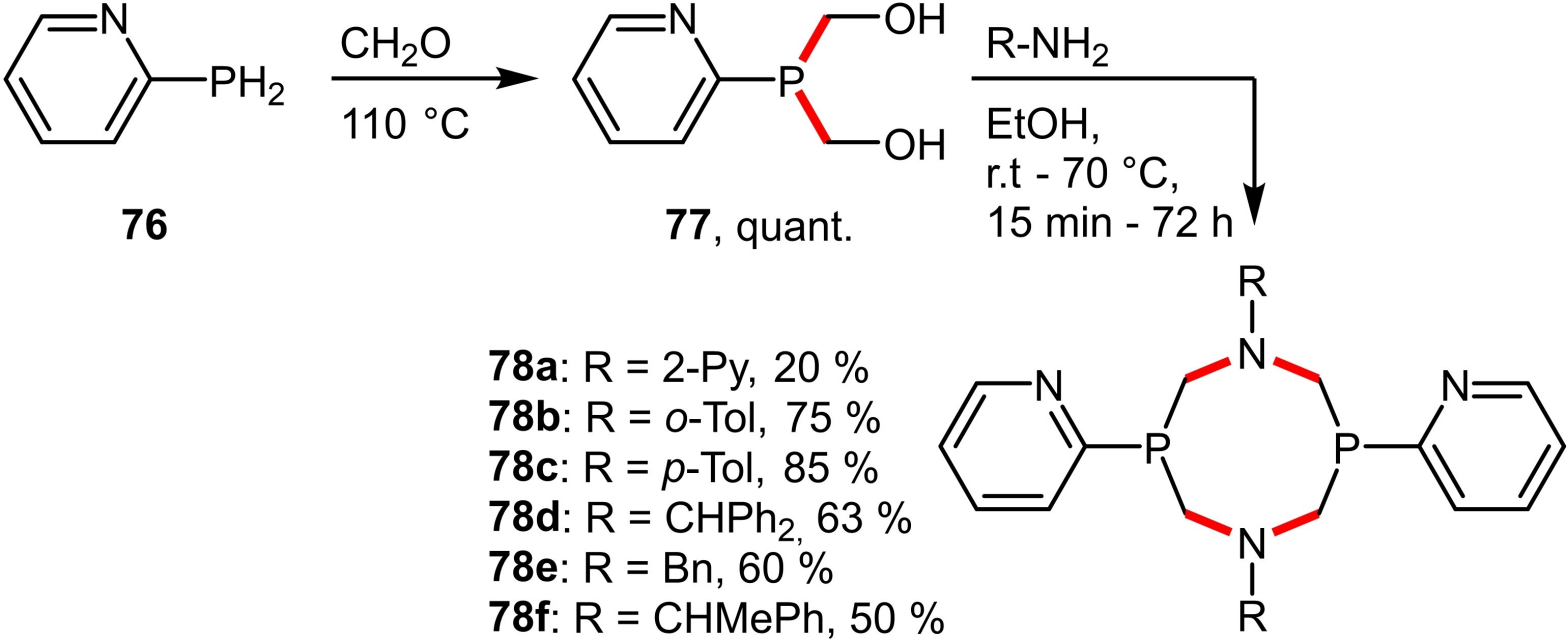
Preparation of 2‐pyridylphosphines based on a cyclic aminomethylphosphine platform.

2‐Pyridyl groups as hydrophilic entities were even more extensively employed in the synthesis of a series of 1,5,3,7‐diazadiphosphacyclooctane ligands (**78 a**–**f**). For this purpose 2‐pyridylphosphine (**76**) was quantitatively bishydroxymethylated and subsequently condensed with various primary amines resulting in air‐stable phosphine ligands in moderate to good yields (Scheme 28). Platinum and palladium complexes of ligands **78 c** and **78 f** were reported to be highly soluble in water.^[65]^

**Scheme 29:**
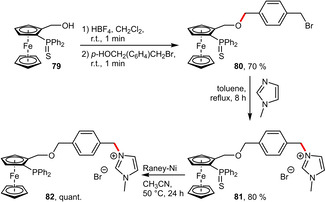
Imidazolium attached to a *p*‐xylene linker.

Alternatively to protonation, alkylation of tertiary amines can also provide positively charged ammonium species. If methylimidazole is attached to a linker bearing an electrophilic benzylic bromide, the resulting product **81** automatically embodies an ionic imidazolium entity (Scheme 29).^[66]^ This strategy based on a *p*‐xylene linker also allowed for the incorporation of neutral hydrophilic groups (Scheme 33) as discussed in the following chapter.

### 2.4 Neutral Hydrophilic Groups

Neutral hydrophilic groups are nonionizable but contain other polar functional groups, which increase the hydrophilicity of the ligand. Recent developments in this field took advantage from hydroxyalkyls, (thio)ureas, polyethylene glycols and phosphonate esters. These groups may either be attached at nucleophilic or electrophilic positions on prefunctionalized ligands.

**Scheme 30:**
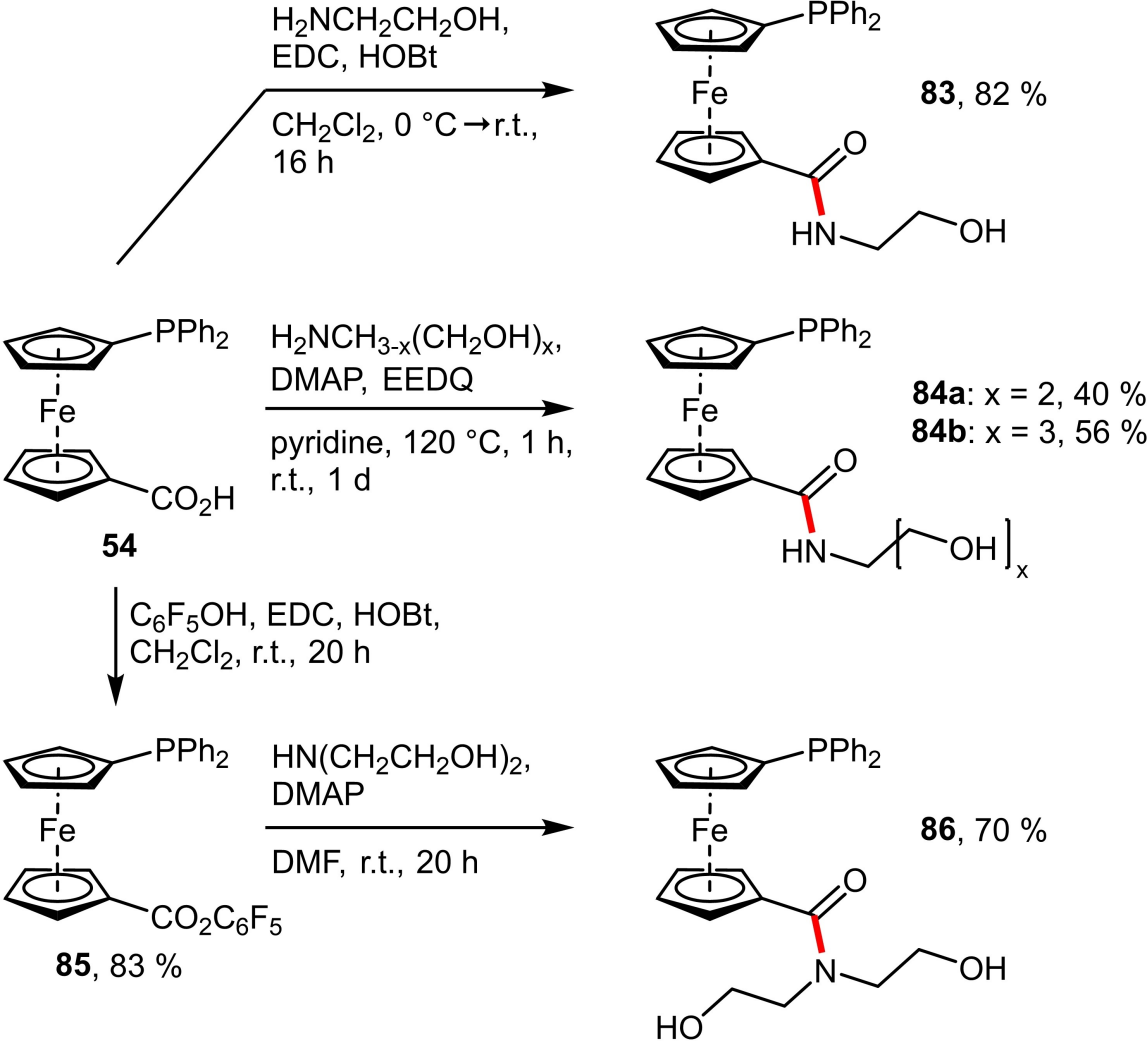
Synthesis of hydroxyalkyl‐substituted ferrocenes.

**Alcohols**. Carboxylic acid groups provide an exceptionally useful handle for the installation of hydrophilic substituents. The group of Štěpnička extensively studied the functionalization of 1’‐(diphenylphosphanyl)ferrocene‐1‐carboxylic acid (Hdpf, **54**) with several polar functional groups. Direct reaction of **54** in presence of common coupling agents afforded mono‐, bis‐ and tris(hydroxymethyl)methanamine derivatives (**83, 84 a, 84 b**) in moderate to good yields (Scheme 30).^[67],[68]^ The corresponding tertiary amide **86**, however, had to be prepared via the pentafluorophenyl active ester **85**. It is noteworthy that **83** proved to be catalytically more useful than **86**, which was found to be hydrolytically less stable.^[67]^

**Scheme 31:**
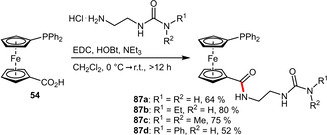
Attachment of urea motifs via an amide linkage.

**(Thio)urea Groups**. The carboxyl group of **54** was also employed to attach urea moieties via an ethylamine linker under classical peptide coupling conditions. This strategy enabled the access to a set of urea‐functionalized phosphinoferrocences (**87 a**–**d**) in moderate to good yields (Scheme 31). Besides the hydrophilic properties, urea motifs induce hydrogen bonding, which is especially pronounced in solid state assemblies.^[69]^

**Scheme 32:**
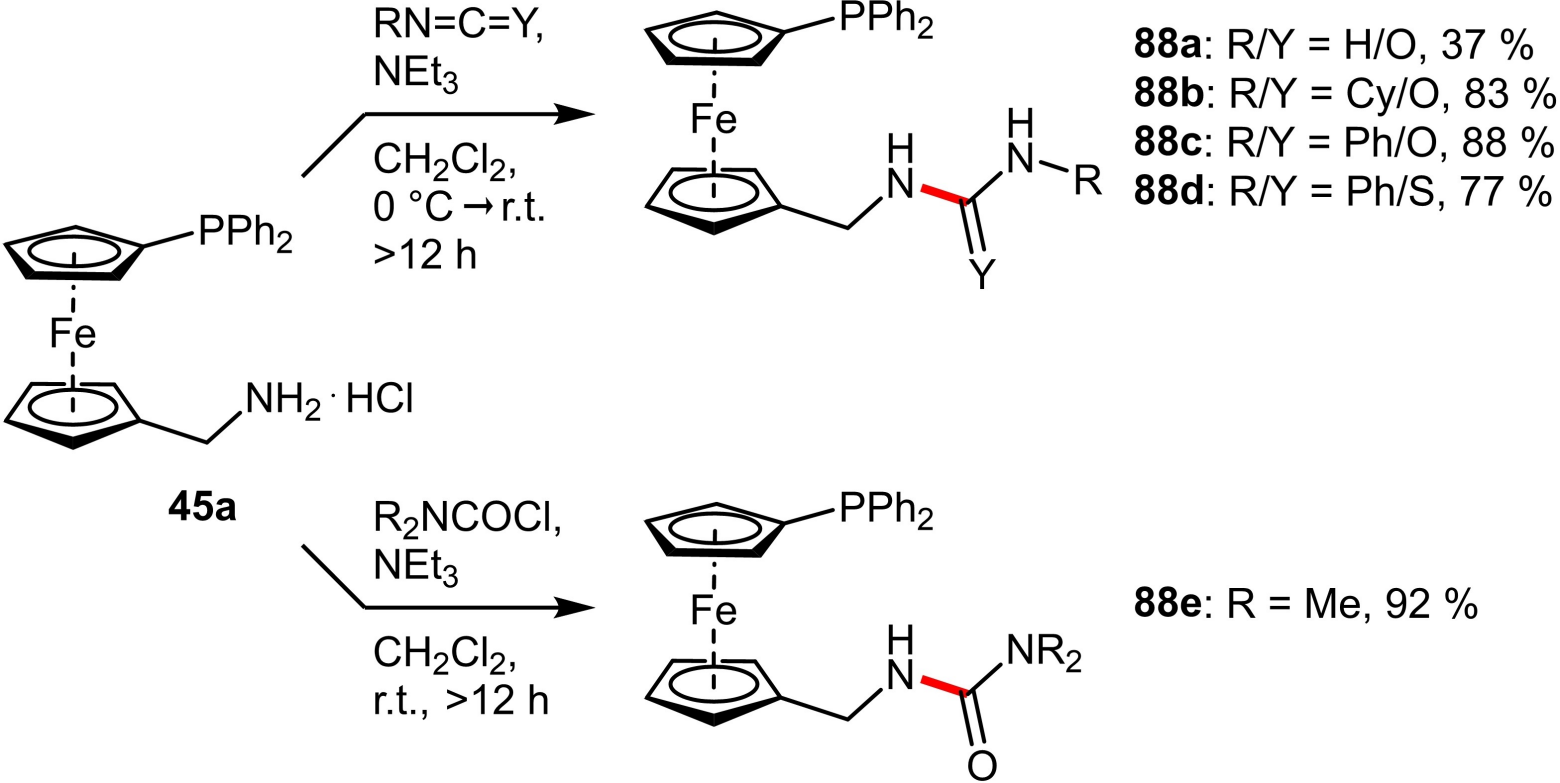
Synthesis of phosphinoferrocenes bearing (thio)urea groups.

Similarily, urea tags can also be readily added to ligands containing free amines such as **45 a**. Depending on the reagent availability, the desired (thio)urea‐tagged ligands (**88 a**‐**e**) were efficiently formed using either iso(thio)cyanate or carbamoyl chloride electrophiles (Scheme 32). The significantly lower yield for **88 a** was attributed to a low equilibrium concentration of HNCO. In addition to its solubilizing function the (thio)urea groups also exhibited hydrogen‐bonded assemblies in the solid state,^[70]^ which might influence the catalytic properties in solution.[^71^, ^72^]

**Scheme 33:**
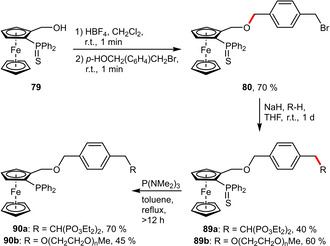
*p*‐Xylene‐based linker for the attachment of hydrophilic groups.

**PEG Groups**. A conceptionally different strategy was developed for the modification of hemilabile and planar chiral *P*,*O*‐ferrocenyl ligands (Scheme 33). Starting from the thio‐protected phosphine **79** (to avoid oxidation), strongly acidic HBF_4_ was used to generate a ferrocenyl carbocation, which can be quenched with nucleophiles. *p*‐(Bromomethyl)benzyl alcohol was found to be very suitable since it incorporates an additional electrophilic handle. This benzylic bromide was readily substituted with hydrophilic entities such as tetraethylmethylenebisphosphonate or monomethylether mPEG 750. Upon desulfurization using P(NMe_2_)_3_ the hydrophilically tagged ligands (**90 a** and **90 b**) were obtained in overall good yield. Importantly, the water‐solubilizing groups are located remotely from the coordinating *P*,*O*‐atoms of the ligand. The hydrophilicity of **90 a** may even be increased by hydrolysis towards the corresponding bisphosphonate salt.^[66]^

**Scheme 34:**
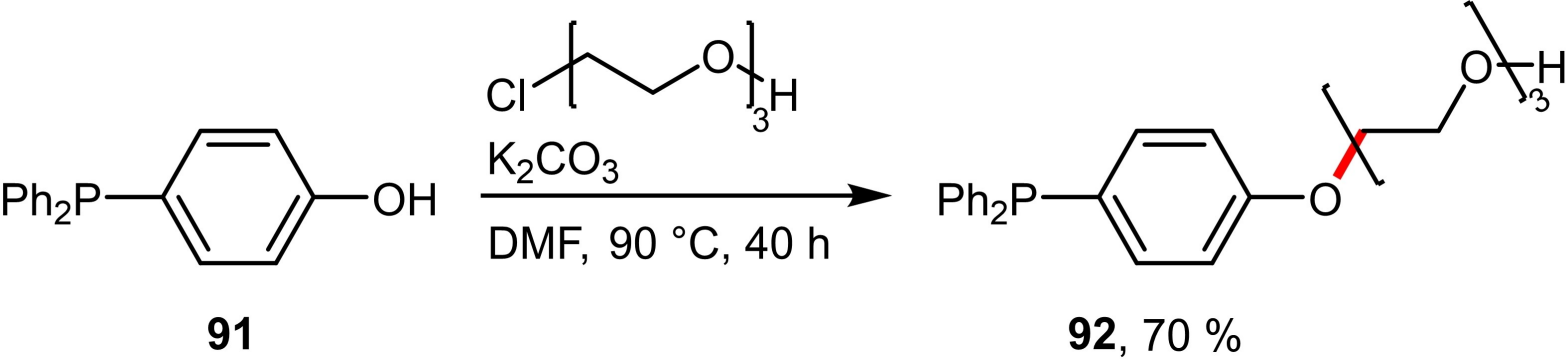
Preparation of TEG‐tagged triphenylphosphine.

Similarly, the phenolic phosphine **91** could be readily functionalized with a triethylene glycol (TEG) group (Scheme 34). This rather short PEG moiety was found to sufficiently increase the water solubility of both the ligand **92** and the resulting ruthenium catalyst in order to enable its application in an aqueous solvent system.^[73]^

**Scheme 35:**
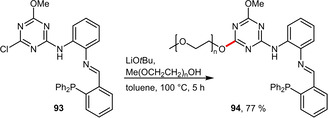
PEGylation of a triazine‐containing ligand.

Another recent example employed a polyethylene glycol in order to transfer the catalytic performance of a triazine based phosphine for carbonylative Heck couplings from organic solvents into water. For this purpose, a mPEG 2000 chain was introduced instead of a methoxy group during the ligand synthesis (Scheme 35).^[74]^

### 2.5 Cyclodextrin‐Based Ligands

Cyclodextrins (CDs) are cyclic oligomers of glucose, which represent an attractive platform for the preparation of water‐soluble phosphine ligands for two main reasons. On the one hand CDs act as neutral, hydrophilic groups due their intrinsic high polarity because of their oxygen‐rich skeleton. Additionally, their cyclic structure provides a cavity, which can function as molecular recognition element enabling binding of the substrate and thus mediating catalysis.

**Scheme 36:**
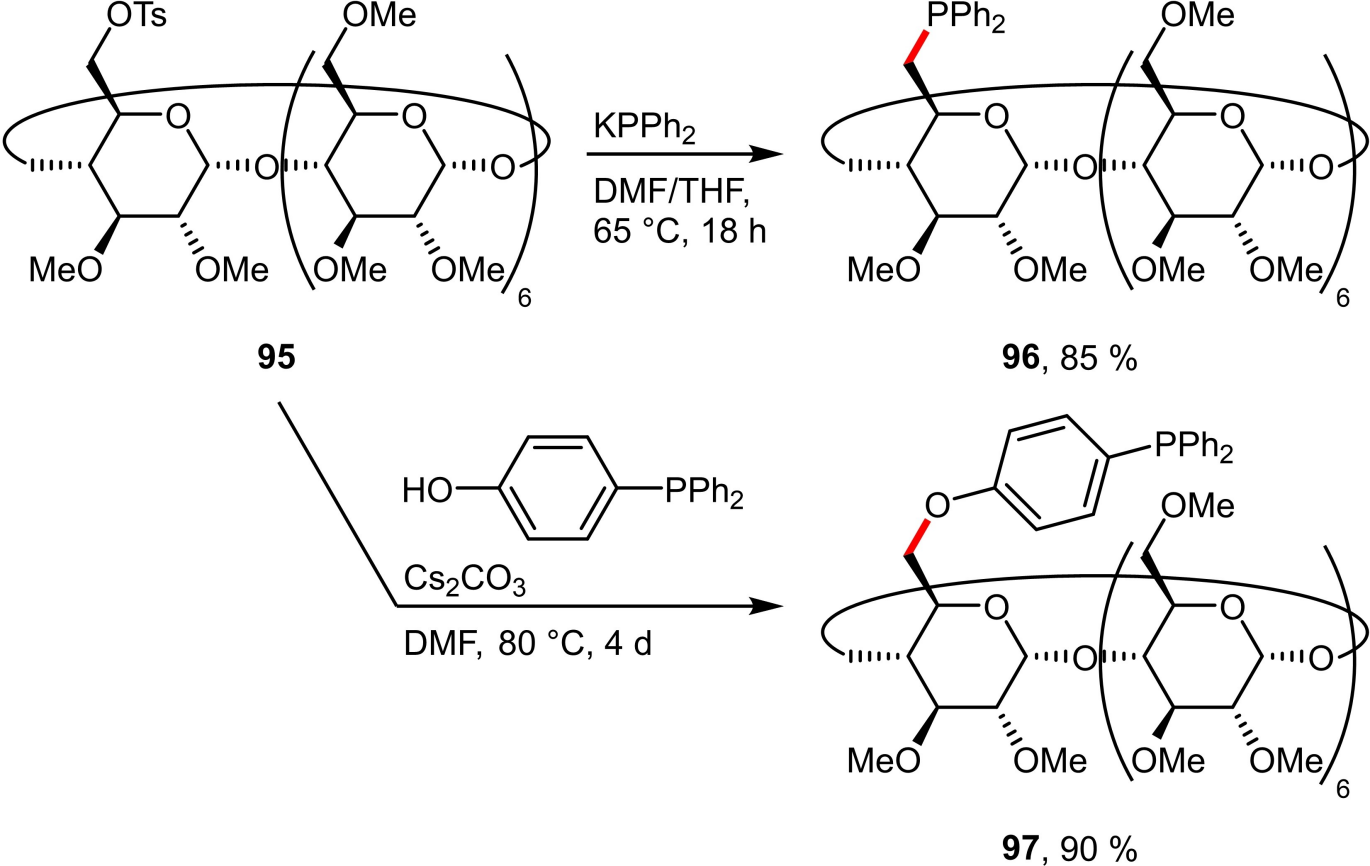
Permethylated β‐CD as platform for di‐ and triarylphosphines.

Most representatives of this class of ligands are functionalized on the sterically more accessible primary site of a single glucose unit. For this purpose, mono‐tosylated CD serves as synthetically especially versatile precursor. The Monflier group prepared the diphenylphosphine **96** by nucleophilic displacement of the tosylate group on a permethylated β‐CD using KPPh_2_ in high yield (Scheme 36). Interestingly, **96** exhibited dual solubility in both organic solvents (MeOH, EtOH, CHCl_3_, CH_2_Cl_2_, THF, DMF, cyclohexane, heptane) and water, spanning an enormous polarity range.^[75]^

Unfortunately, **96** tends to self‐inclusion of a phenyl group into the CD cavity and thus hampers its recognition and catalytic properties. In order to prevent this phenomenon, a follow‐up study envisioned a rigid phenylene group between the CD and the phosphine. Starting from the same substrate **95**, the modified triarylphosphine ligand **97** could be efficiently obtained (Scheme 36). Although this ligand was only slightly soluble in water (17 mg L^−1^), the resulting rhodium complexes were substantially soluble (2.6 to 9.1 g L^−1^).^[76]^

**Scheme 37:**
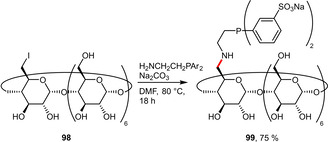
Sulfonated diphenylphosphine linked via a C2‐spacer to β‐CD.

Following a similar approach, mono‐iodide β‐CD (**98**) was treated with an aminoethyl‐containing phosphine precursor resulting in the attachment of the *P*‐donor via a linker to the β‐CD platform (Scheme 37). The ligand **99** was afforded in good yield and was significantly more water‐soluble (63 g L^−1^) than **97** due to the sulfonated phenyl substituents.^[77]^

**Scheme 38:**
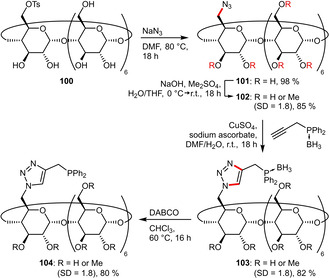
Multi‐step protocol for the click‐attachment of a phosphine substituent to RAME‐β‐CD.

Another ligand of this class was based on randomly methylated CD (RAME‐CD) due to their intrinsically higher water‐solubility. The multi‐step synthesis originates again from mono‐tosylated CD **100**, which was first converted to its azide **101** and then partially methylated, resulting in an average degree of substitution (DS) of 1.8 per glucose unit. Subsequently, the azide group was engaged in a CuAAC to attach the borane‐protected phosphine, which was deprotected in the final step using DABCO to afford the desired ligand **104** in overall high yield (Scheme 38).^[78]^

### 2.6 PTA‐Based Ligands

*P*‐Donor ligands derived from the cage‐like structured 1,3,5‐triaza‐7‐phosphaadamantane (PTA) do not only provide a neutral‐polar environment to enable water‐solubility but already incorporate the *P*‐donor atom. Even though the parent compound PTA has been known for many years,^[79]^ it still represents an attractive platform for the development of new ligand and catalyst systems.

**Scheme 39:**
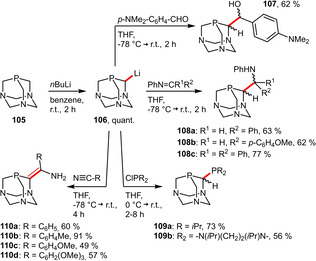
Overview of recent advances in the preparation of PTA‐based phosphine ligands.

A vast majority of PTA‐based derivatives is accessible from the upper‐rim lithiated species **106**. The protocol for the preparation of this valuable intermediate was recently improved.^[80]^ This precursor can then be treated with various electrophiles to install a second donor atom in order to allow for a hemilabile, bidentate coordination behaviour to metals (Scheme 39).

The β‐hydroxyphosphine **107** was synthesized by the reaction with *p*‐dimethylaminobenzaldehyde in good yield as a separable mixture of diastereomers. **107** was reported to be moderately water‐soluble (1.9 g L^−1^).^[80]^

When **106** is treated with imines, β‐aminophosphine ligands **108 a**–**c** are obtained as diastereomeric mixtures, which can be separated by repeated crystallization. Compounds **108 a**–**c** feature slightly higher water‐solubilities (2.7–4.9 g L^−1^) and ruthenium complexes of ligands **108 a** and **108 b** are soluble as well (6 and 5 g L^−1^, respectively).^[81]^

Bidentate ligands **109 a** and **109 b** containing two *P*‐donors could be prepared by treatment of **106** with the corresponding ClPR_2_ precursors. Although ligand **109 a** was highly air‐sensitive it was somewhat soluble in water (13.3 g L^−1^). Moreover, two ruthenium complexes of **109 a** were soluble in water as well. However, its phosphinediamine derivative was found to decompose in water, presumably by hydrolysis of the P−N bonds.^[82]^

Most recently, the group of Frost explored the reaction of **106** with several aromatic nitriles leading to β‐phosphinoenamines **110 a**–**d** in modest to excellent yields. All of the prepared ligands exhibited reasonably water‐soluble properties (**110 a**: 4.0 g L^−1^, **110 b**: 4.2 g L^−1^, **110 c**: 3.7 g L^−1^, **110 d**: 4.7 g L^−1^).^[83]^

## 3 Recent Catalytic Applications of Hydrophilic Phosphorus Ligands

In this section catalytic applications of water‐soluble phosphine ligands, which have been reported since the seminal review by Shaughnessy,^[12]^ are described. Therefore, novel ligands, which have been discussed in the preceding chapters, as well as established hydrophilic phosphines are included.

### 3.1 Hydroformylation

Hydroformylation of olefins is among the most important reactions performed on a multi‐ton scale. It is moreover the most popular industrial application of hydrophilic phosphine ligands since the development of the renowned Ruhrchemie‐Rhône Poulenc process. Although the most commonly used ligand TPPTS exhibits excellent coordination ability and solubility in water, there is still an ongoing effort to establish hydroformylation reactions based on novel ligands to expand the scope to higher olefins, which are otherwise troublesome.^[1]^

**Scheme 40:**
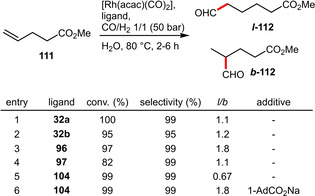
Rhodium‐catalyzed hydroformylation of methyl 4‐pentenoate.

Recent reports of hydroformylation under aqueous conditions uniformly focus on phosphine ligands that are either sulfonated or grafted on cyclodextrins (CDs) to enhance their solubility in water. The performance of the sulfonated phosphines (**32 a**, **32 b**) and CD‐derived phosphines (**96**, **97**, **104**) in the hydroformylation of the moderately hydrophilic substrate methyl 4‐pentenoate (**111**) is summarized in Scheme 40. The structurally closely related ligands **32 a** and **32 b** led to very high conversion as well as excellent chemoselectivity for aldehydes but suffered from virtually no regioselectivity.^[39]^ Application of the CD‐grafted ligand **96** resulted in a significantly more regioselective outcome (*l*/*b*=1.8), while retaining high conversion and selectivity.^[75]^ In contrast the more rigid derivative **97** was found to be inferior for this substrate.^[76]^ In the case of ligand **104** the *l*/*b* ratio could be inverted from 0.67 to 1.8 in presence of sodium 1‐adamantanecarboxylate (1‐AdCO_2_Na, 3 eq. with respect to **104**). This can be explained by the inclusion of this guest molecule in the cavity of **104** inducing a conformational change.^[78]^

**Scheme 41:**
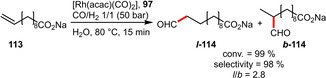
Rhodium‐catalyzed hydroformylation of sodium 10‐undecenoate.

Despite the fact that ligand **97** only exhibited poor regioselectivity in the case of methyl 4‐pentenoate (**111**), it performed significantly better with the longer‐chain substrate **113** (Scheme 41). In this case the olefin can form an inclusion complex with **97** resulting in a drastically increased *l*/*b* ratio as well as TOF (1980 h^−1^ compared to 205 h^−1^).^[76]^ This showcases that a recognition motif in vicinity to the catalytic site can significantly improve the catalytic performance.

**Scheme 42:**
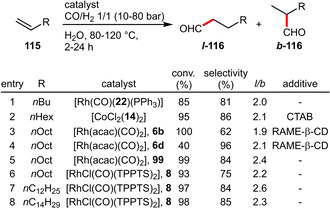
Rhodium‐/cobalt‐catalyzed hydroformylation of olefins.

Very recently the group of Štěpnička reported the hemilabile *P*,*O*‐chelate **22**, which could be successfully employed in the hydroformylation of 1‐hexene (Scheme 42).^[84]^ Another study was published on the Co‐catalyzed hydroformylation of 1‐octene with special attention on different mass transfer promoters and their influence on conversion, chemo‐ as well as regioselectivity.^[85]^ Monflier et al. systematically replaced the *m*‐sulfonated phenyl groups in TPPTS with either 1‐ or 2‐naphthyl groups resulting in a set of different ligands (**6 a**–**f**), which were subjected to Rh‐catalyzed hydroformylation of 1‐decene. Key findings indicated that (i) the conversion could be improved by RAME‐β‐CD as mass transfer agent and (ii) the sterically more congested 1‐naphtyl‐substituted ligands (e. g. **6 b**) resulted in higher conversions even though at the expense of lower selectivities and more catalyst leaching.^[25]^ A more recent report by the same group introduced the CD‐derived ligand **99** achieving overall better performance including some catalyst robustness in recycling experiments. This catalyst system also showed promising results for even longer and thus more hydrophobic terminal olefins.^[77]^ In comparison, the application of the sulfonated triarylphosphine **8** bearing a long chain alkyl group was especially useful for higher olefins. Due to the dual role of **8** acting as both phosphine ligand and micelle‐forming agent, even 1‐tetradecene and 1‐hexadecene could be converted after prolonged reaction times (22 h instead of 2 h for 1‐decene). The poor recyclability of catalysts derived from **8** was attributed to phosphine oxidation rather than leaching to the organic phase.^[27]^

**Scheme 43:**
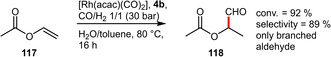
Rhodium‐catalyzed hydroformylation of vinyl acetate.

In addition, comparable Rh‐catalysts have also been reported for the hydroformylation of functionalized olefins such as vinyl acetate (Scheme 43). Trifluoromethyl‐substituted triphenylphosphines (**4 b** in particular) were found to be superior in terms of conversion and TOF over the related TPPMS, TPPDS and TPPTS due to their increased π‐accepting capabilities. Catalyst recycling could be realized for up to 4 runs without significantly diminished conversions.^[24]^

### 3.2 Hydrogenation

**Scheme 44:**
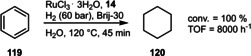
Ruthenium‐catalyzed hydrogenation of benzene.

The hydrogenation of benzene is probably the most fundamental C=C hydrogenation reaction, which is conducted annually on a multimillion‐ton scale. In order to alleviate the need for drastic conditions, the Monflier group was able to show that a Ru‐catalyst derived from the sulfonated tris(biphenyl)phosphine **14** could provide a viable alternative (Scheme 44). The conversion and TOF was especially good in presence of the non‐ionic surfactant (Brij‐30) due to an increase of the aqueous solubility of the substrate. Recycling studies indicated a slight decrease in conversion and TOF already over the first four runs.^[30]^

**Scheme 45:**
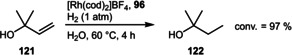
Rhodium‐catalyzed hydrogenation of 2‐methyl‐3‐buten‐2‐ol.

The CD‐based ligand **96** was reported to be capable of catalyzing the hydrogenation of the allylic alcohol **121** under low‐pressure conditions (Scheme 45). It is noteworthy that **121** did not form an inclusion complex with the CD indicating that it is solely functioning as ligand. Due to the amphiphilic character of **96** (*P*_ow_=0.48) and the resulting solubility in organic solvents, catalyst recycling is precluded.^[75]^

**Scheme 46:**
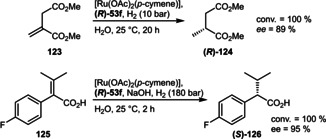
Asymmetric ruthenium‐catalyzed hydrogenation of α,β‐unsaturated carboxylates.

Hydrophilic MeOBIPHEP derivatives featuring axial chirality could be employed in asymmetric hydrogenation of α,β‐unsaturated carboxylic substrates exhibiting high reactivities and enantioselectivities (Scheme 46). The utility was demonstrated on the preparation of a key intermediate (**126**) in the synthesis of the active pharmaceutical ingredient (API) Mibefradil.^[51]^

**Scheme 47:**
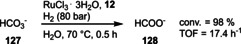
Ruthenium‐catalyzed hydrogenation of bicarbonate.

During the past years, a considerable interest in formic acid as hydrogen source and storage material has emerged.^[86]^ In a recent study, the key process of this concept, namely the Ru‐catalyzed hydrogenation of bicarbonate to formate (Scheme 47), was investigated using various sulfonated triarylphosphines showing TOF up to 17.4 h^−1^. Although TOF values remained constant over the first three runs, protection against air was crucial.^[87]^

**Scheme 48:**
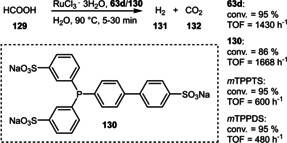
Ruthenium‐catalyzed dehydrogenation of formic acid.

The reverse reaction liberating hydrogen can be efficiently catalyzed by similar Ru‐complexes. These reactions were found to reach the maximal conversion (ca. 90 %) much faster, namely within a few minutes (Scheme 48). In comparison to the benchmark ligand TPPTS (**12**), more basic and therefore stronger σ‐donating ligands like **130** were even more active (TOF up to 1668 h^−1^) and could be recycled in eleven consecutive runs without loss of catalytic activity.^[88]^ In contrast to the negatively charged **130**, the cationic ligand **63 d** was similarly active surpassing comparable sulfonated ligands (e. g. *m*TPPTS, *m*TPPDS). This was attributed to a faster coordination of anionic species (HCOO^−^, HCO_3_^−^, H^−^). Noteworthily, a catalyst derived from the related phosphine **63 c** could be recycled for more than 30 runs with a total TON exceeding 10000.^[56]^

### 3.3 Allylation

**Scheme 49:**
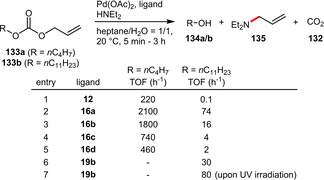
Palladium‐catalyzed cleavage of allyl alkyl carbonates.

Allylic carbonates can be very effectively activated by various Pd‐catalysts. The Monflier group assessed several of their hydrophilic phosphines in the biphasic Pd‐catalytic cleavage of allyl alkyl carbonates using diethylamine as scavenger (Scheme 49). All of the tested ligands (**16 a**–**d** and **19 b**) were found to be significantly more active than TPPTS (**12**). This trend was especially pronounced for the more hydrophobic allyl undecyl carbonate (**133 b**) due to the amphiphilic character of the ligands **16 a**–**d**.^[32]^ It is worth mentioning that in the case of **16 a** and **16 b** stable emulsions precluded the recyclability of the catalysts. The diazo‐containing phosphine **19 b** was demonstrated to exhibit a light inducible behaviour. The more stable *trans*‐isomer could be partially isomerized upon UV irradiation leading to the formation of new aggregates having a better ability to solubilize the substrate in water, which resulted in a higher activity.^[33]^

**Scheme 50:**
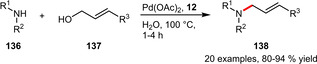
Palladium‐catalyzed amination of allylic alcohols.

Another contribution in this field focused on the direct amination of allylic alcohols without the need for leaving groups or co‐catalysts. Optimization of the reaction conditions enabled the use of just 0.2 mol% Pd(OAc)_2_/TPPTS. The scope involves mainly aromatic amines together with allyl or cinnamyl alcohol affording generally excellent yields (Scheme 50). In addition, the catalyst could be recycled in up to eight runs with almost no decrease in yield.^[89]^

### 3.4 Cross‐Coupling

Transition metal‐catalyzed cross‐coupling reactions are undoubtedly among the most important tools for C−C bond formations. It is therefore not surprising that the use of hydrophilic phosphine ligands in order to enable this type of reaction in aqueous media has been extensively studied. In fact, cross‐coupling reactions account for the largest fraction of applications of water‐soluble phosphines in the recent past.

**Scheme 51:**
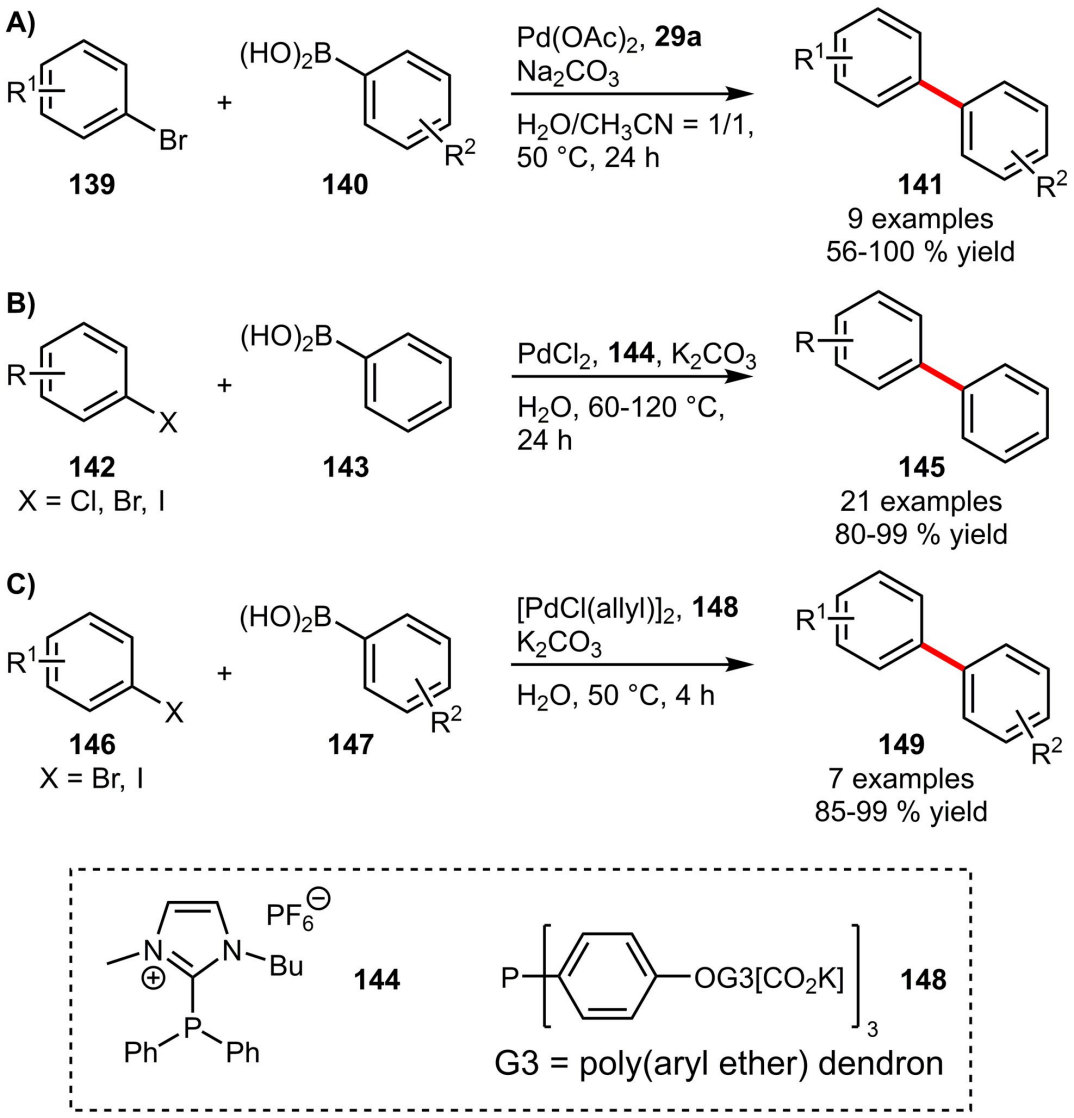
Suzuki‐Miyaura cross‐coupling of arylboronic acids with aryl halides.

**Suzuki‐Miyaura Couplings**. Shaughnessy et al. could show that the novel sulfonated benzylphosphine **29 a** can be applied in the coupling of several arylboronic acids with aryl bromides in a solvent mixture of H_2_O/CH_3_CN=1/1 (Scheme 51A). Interestingly in pure water generally higher yields were obtained.^[38]^ In comparison the imidazolium‐based phosphine **144** exhibited a broader scope for electrophiles including aryl iodides and aryl chlorides (Scheme 51B). The lower reactivity of chloride substrates could be compensated by a higher reaction temperature affording various biaryls in excellent yields. It is noteworthy that the catalyst‐containing aqueous phase could be recycled for six consecutive runs resulting in just a small decrease in yield.^[90]^ The dendritic ligand **148** again was limited to aryl iodides and aryl bromides (Scheme 51C).^[91]^

**Scheme 52:**
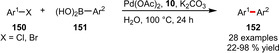
Suzuki‐Miyaura cross‐coupling of (hetero)arylboronic acids with (hetero)aryl halides.

The recently reported disulfonated aryl dialkyl monophosphine **10** proved to be far more general. The corresponding Pd‐catalyst was able to couple a plethora of (hetero)aryl halides (including chlorides) with boronic acid substrates (including moderately hindered ones) in mostly good to excellent yields (Scheme 52). The high catalytic performance is reflected by a TON of 10400 (for naphth‐1‐ylboronic acid and *p*‐acetylchlorobenzene).^[28]^

**Scheme 53:**
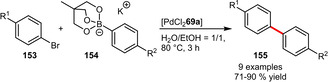
Suzuki‐Miyaura cross‐coupling of triolborates with aryl bromides.

Štěpnička et al. reported a Pd‐catalyst based on the ferrocene ligand **69 a** for the base‐free cross‐coupling of aryl borates in aqueous solvents (Scheme 53). Both electron‐rich and electron‐poor aryl bromides were converted in high yields.^[59]^

**Scheme 54:**
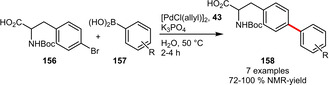
Suzuki‐Miyaura cross‐coupling of arylboronic acids with a *N*‐protected amino acid.

The *N*‐protected amino acid **156** could be successfully engaged in a Suzuki‐Miyaura reaction using ligand **43** with several arylboronic acids in water (Scheme 54). No deprotection of the *N*‐protecting group was observed under the reported conditions.^[42]^

**Scheme 55:**
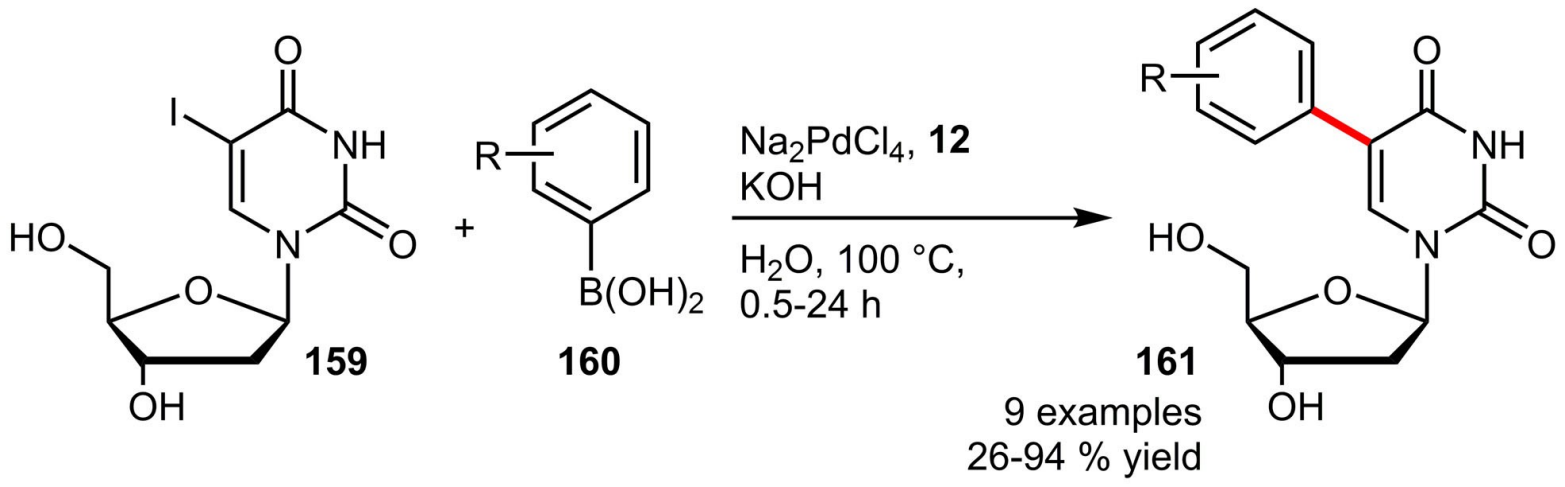
Suzuki‐Miyaura cross‐coupling of arylboronic acids with 5‐iodo‐2’‐deoxyuridine.

In the context of biologically relevant substrates, nucleosides have also been considered as demonstrated by the Suzuki‐Miyaura arylation of 5‐iodo‐2’‐deoxyuridine employing a TPPTS‐based Pd‐catalyst (Scheme 55). This approach allowed the incorporation of several functionalized arylboronic acids at a low catalyst loading of 0.1 mol%.^[92]^ In addition a similarly active system was reported using a PTA‐based catalyst.^[93]^

**Scheme 56:**
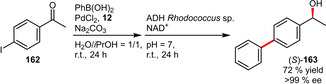
Chemoenzymatic one‐pot process involving a Suzuki‐Miyaura reaction followed by a biocatalytic reduction.

Gröger et al. published a chemoenzymatic one‐pot process combining a Pd/TPPTS‐catalyzed cross‐coupling and a biocatalytic reduction (Scheme 56). By careful selection of the reaction conditions it could be ensured that both reactions can proceed in the same solvent and are not inferring with each other. The resulting alcohol **163** could be isolated in good yield and excellent enantioselectivity.^[94]^

**Scheme 57:**
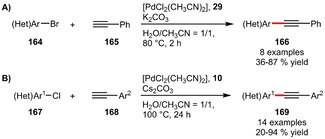
Sonogashira coupling of aryl halides.

**Sonogashira Couplings**. Next to Suzuki‐Miyaura cross‐couplings, hydrophilic phosphines have also been successfully employed in Sonogashira reactions. Ligand **29** enabled the coupling of phenyl acetylene with several (hetero)aryl bromides in moderate to good yields (Scheme 57A).^[38]^ In presence of the phosphine **10** an even broader range of substrates (including (hetero)aryl chlorides) was tolerated in mostly excellent yields (Scheme 57B).^[28]^

**Scheme 58:**
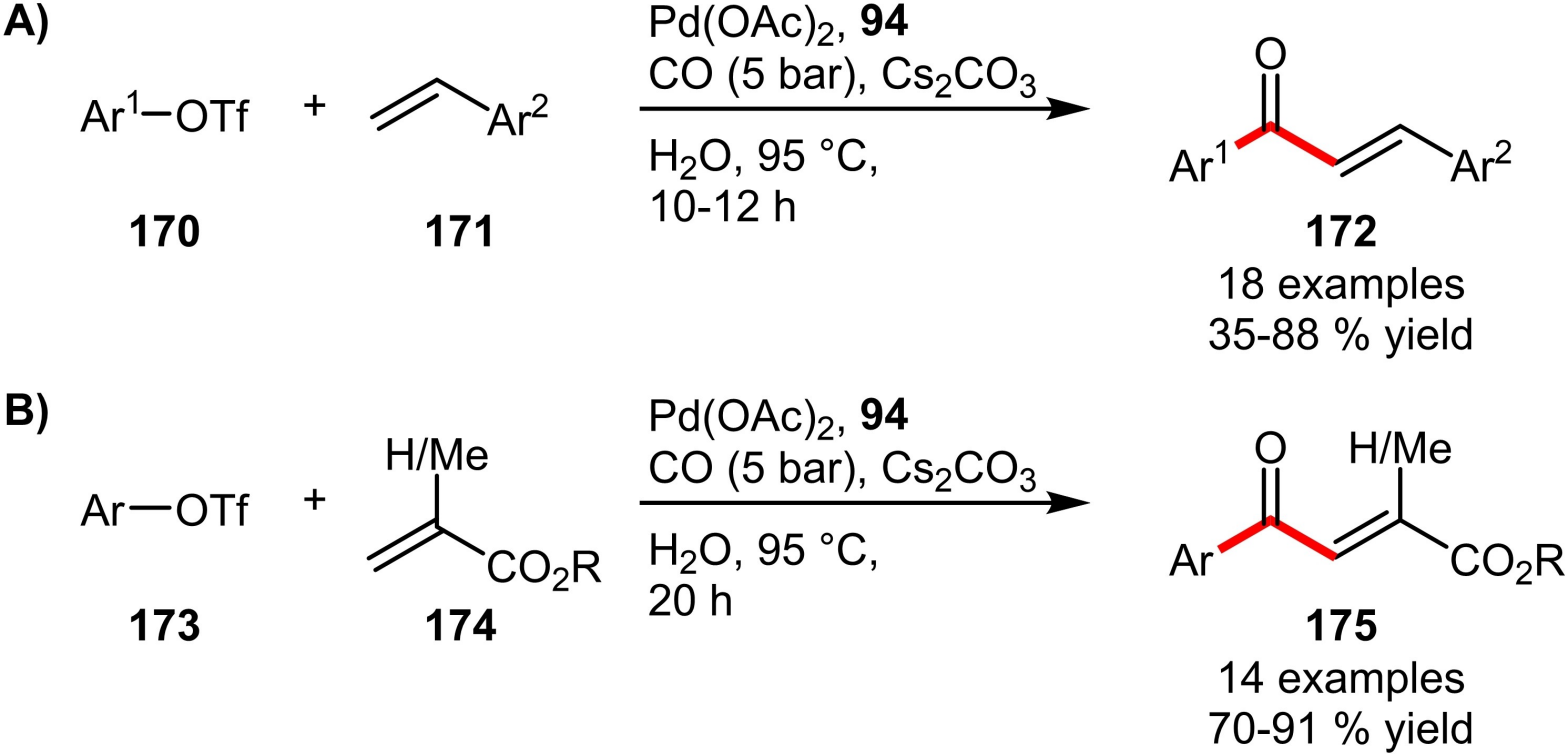
Palladium‐catalyzed carbonylative Heck reaction.

**Heck reactions**. Carbonylative Heck reactions in water could be realized by means of the PEG‐modulated s‐triazine‐based phosphine **94**. Starting from either styrenes (Scheme 58A) or acrylates (Scheme 58B) a diverse set of α,β‐unsaturated ketones could be prepared in mostly high yields. Poorer yields, however, were obtained in the case of aryl triflates bearing strong electron‐withdrawing groups. Moreover, the Pd‐catalyst in the carbonylative Heck coupling could be reused for five times although suffering from decreasing yields.^[74]^

**Scheme 59:**
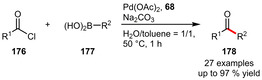
Palladium‐catalyzed cross‐coupling reaction of arylboronic acids with benzoyl chlorides.

**Other Couplings**. Water‐soluble phosphines derived from ferrocene proved especially useful for the Pd‐catalyzed arylation of benzoyl chlorids affording ketones as reported by the group of Štěpnička. The catalytic system based on ligand **68** for example exhibited good functional group tolerance giving the desired ketones in moderate to very high yields (Scheme 59).^[58]^ Similar results were obtained using ligands **46** ^[45]^ and **22**.^[34]^ In general the best yields were achieved when electron‐rich boronic acids are reacted with electron‐poor benzoyl chlorides.^[34],[45]^ Recently, a related catalyst system derived from ligands **69 b**–**d** was introduced for CF_3_‐substituted derivatives.^[60]^

**Scheme 60:**
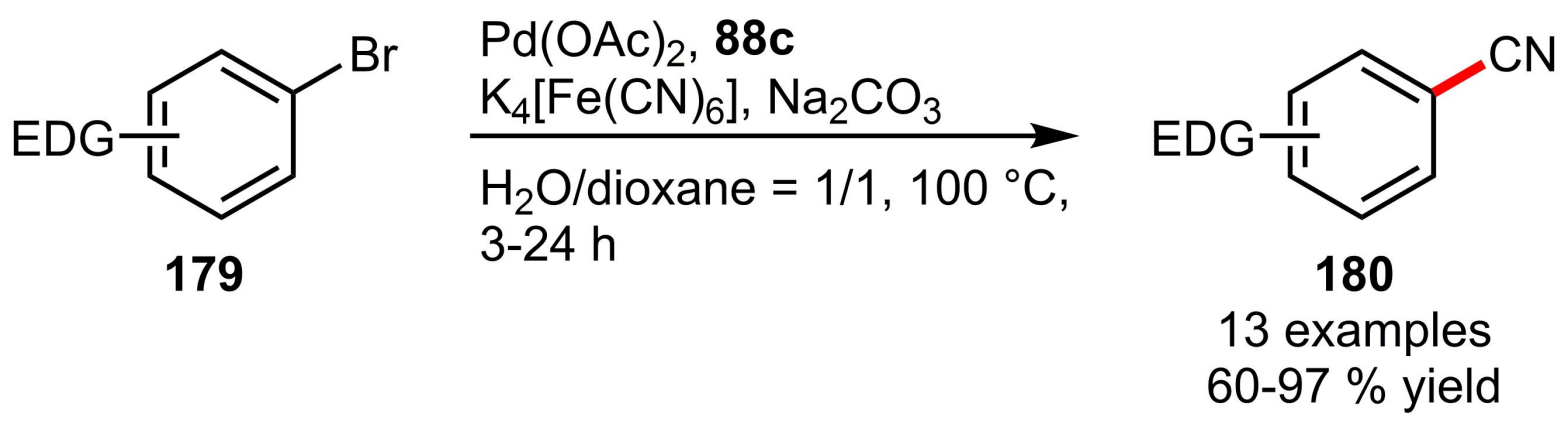
Palladium‐catalyzed cyanation of aryl bromides.

Štěpnička et al. could also apply hydrophilic phosphines such as **88 c** for the cyanation of aryl bromides in aqueous solvents (Scheme 60). This reaction proved especially useful for electron‐rich substrates, while electron‐poor ones (e. g. 4‐Ac, 4‐CF_3_, 4‐Cl, 4‐NO_2_) suffered from low conversion and/or side reactions.^[70]^ Also the sulfonated phosphines **43** ^[41]^ and **44 a** ^[43]^ exhibited similar trends. Notably, solvent mixtures with similar shares of water and organic solvent performed significantly better than pure solvents.^[43],[70]^

### 3.5 Hydration of Nitriles

Primary amides are frequently encountered intermediates in the production of pharmaceutical, agricultural and other fine chemicals. However, direct hydrolysis of nitriles to primary amides still represents a challenge since chemical hydrolysis by strong acid or base catalysts suffers from a low functional group tolerance or undesired formation of carboxylic acids, while biocatalysts usually show a narrow substrate specificity. For this reason, a transition metal‐catalyzed transformation in water seems highly attractive.^[63]^

**Scheme 61:**
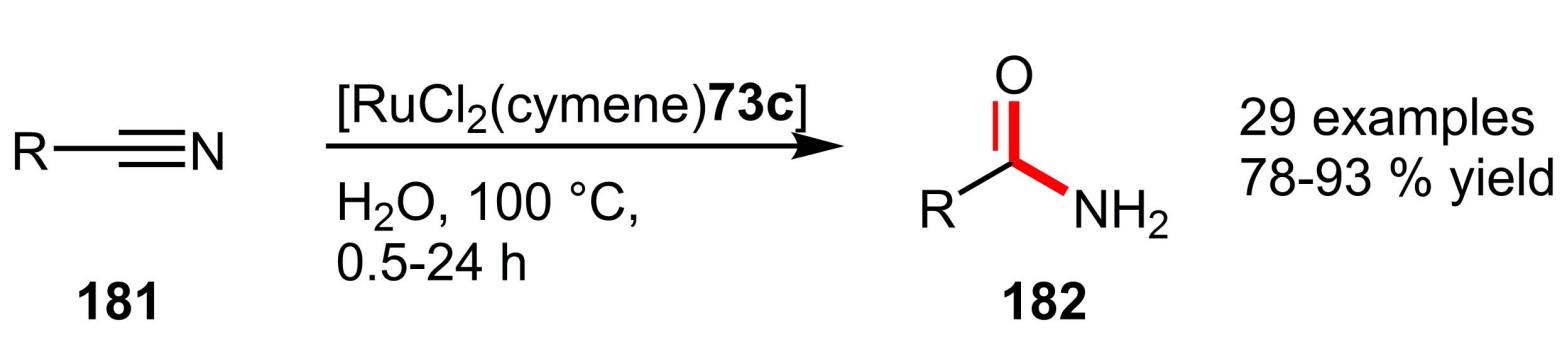
Ruthenium‐catalyzed hydration of nitriles to amides.

To this end, several Ru‐catalyzed reactions employing hydrophilic phosphines have been reported. The thiazolium‐containing ligand **73 c** was found to be especially useful exhibiting a broad substrate scope including both aryl and alkyl nitriles, α,β‐unsaturated substituents and potentially hydrolyzable ester groups, affording primary amides in very good yields (Scheme 61). The catalytic performance was characterized by TON up to 9800, operability at mild temperatures (40 °C) and recyclability for five consecutive cycles. Although high conversions were observed until the fifth run, the activity was diminished due to incomplete catalyst recovery.^[63]^

Additionally, PTA‐based Ru‐catalysts derived from ligands **108 c**,^[81]^ **109 a**,^[82]^ and PTA^[95]^ itself were reported to operate under comparable conditions. It is worth mentioning that [RuCl(toluene)**108c**]Cl was extremely active exhibiting TON and TOF of up to 97000 and 285 h^−1^, respectively.^[81]^ On the other hand, [RuCl_2_(PTA)_4_] could be recycled for up to seven runs without any decrease in activity and selectivity.^[95]^

### 3.6 Other Applications

**Scheme 62:**
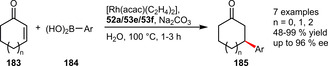
Rhodium‐catalyzed 1,4‐addition of arylboronic acids to enones.

An enantioselective conjugate addition reaction in water was described using a Rh‐catalyst based on atropisomeric MeOBIPHEP analogues **52 a**, **53 e**, and **53 f**. By careful selection of the ligand, arylboronic acids could undergo 1,4‐addition to cyclopentenone, cyclohexenone and cycloheptenone affording the desired products in moderate to excellent yields and enantioselectivities (Scheme 62).^[96]^

**Scheme 63:**
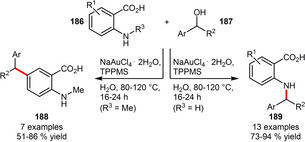
Gold‐catalyzed *C*‐ or *N*‐benzylation of anthranilic acids.

The monosulfonated triphenylphosphine TPPMS can be engaged in an Au‐catalyzed benzylation of anthranilic acid derivatives in water using environmentally more benign benzylic alcohols as electrophiles. Under identical conditions benzylation took place chemoselectively either at the aromatic ring (*N*‐methyl anthranilic acids) or at the *N*‐atom (unsubstituted anthranilic acids). Both classes of products were obtained in good to high isolated yields (Scheme 63).^[97]^

**Scheme 64:**
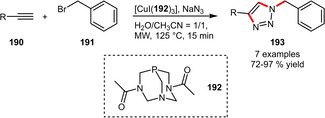
Copper‐catalyzed three‐component azide‐alkyne click reaction.

Very recently, the PTA‐type ligand **192** was reported for a three‐component CuAAC reaction starting from benzyl bromide, sodium azide and several alkynes (Scheme 64). The corresponding 1,4‐disubstituted 1,2,3‐triazoles were obtained in good to excellent yields after only 15 min of reaction time under microwave irradiation. Although the reaction required an organic co‐solvent to give high conversions, the hydrophilic catalyst system offers the possibility of being easily separable by washing with water.^[98]^

**Scheme 65:**
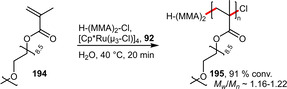
Aqueous living radical polymerization of poly(ethylene glycol) methacrylate (PEGMA).

The TEGylated triarylphosphine **92** could be employed in a Ru‐catalyzed living radical polymerization of PEGMA (**194**) in water using H−(MMA)_2_−Cl as initiator (Scheme 65). A conversion of 91 % could be observed after only 20 min. Therefore, this catalyst system was found to be more active and exhibited a narrower molecular weight distribution (MWD) in comparison to the non‐TEGylated ligand.^[73]^

## 4 Conclusion

In this review recent efforts in the synthesis of hydrophilic phosphines employing water‐solubilizing functional groups (anionic, cationic, and neutral) were described. Although the iconic TPPTS ligand has been known for decades, the preparation of water‐soluble phosphorus ligands still represents a major synthetic challenge, especially in the case of sensitive ligands such as hydrolytically labile phosphites. Despite the prolific advancements in the field of transition metal‐catalyzed reactions in aqueous media, the number of commercially available ligands is still small,^[99]^ fueling the need for the synthesis of new hydrophilic ligands.

In our opinion, the development of novel ligands has not kept up with the demand posed by the wide variety of possible applications, some of which were highlighted in this review. We are thus convinced that especially the development of new reliable solubilization strategies would spark a large number of new catalytic applications. Next to environmentally beneficial aspects, the combination of metal‐ and biocatalysts as well as the modification of biomolecules (e. g. proteins) by metal catalysis would profit tremendously from new developments in this field.

## Biographical Information

*Thomas Schlatzer was born in 1994 in Voitsberg (Austria). He completed his undergraduate studies in Chemistry at the Graz University of Technology and the University of Graz. In 2017, he received his MSc degree working on the synthesis of water‐soluble phosphites. He then continued in the group of Prof. Rolf Breinbauer at the Graz University of Technology pursuing his PhD studies focused on the development of bioorthogonal protein modifications via Pd‐catalysis*.

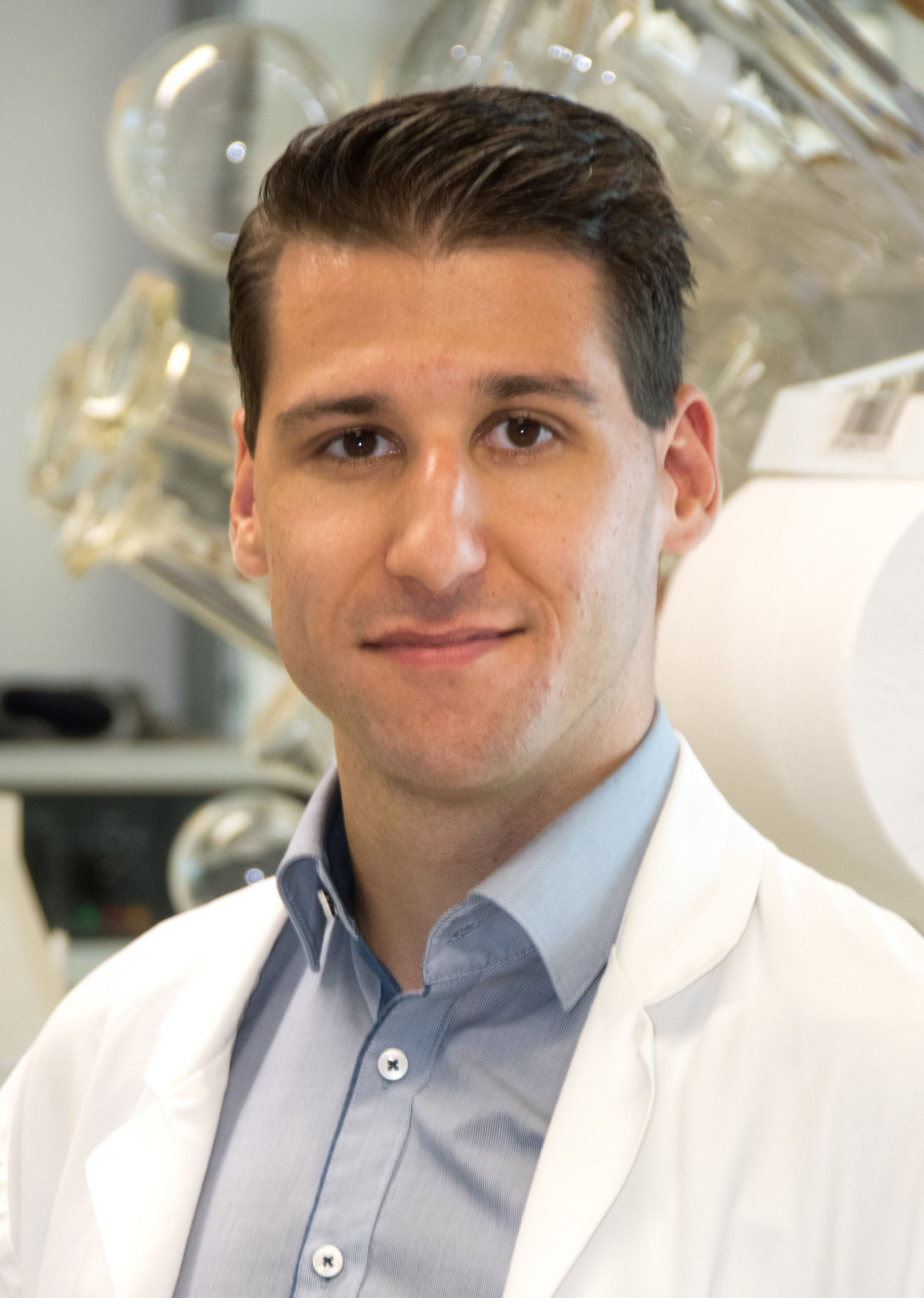

## Biographical Information

*Rolf Breinbauer was born in 1970 in Schärding (Austria). He studied Chemistry in Vienna and Heidelberg and received his PhD at the MPI für Kohlenforschung in Mülheim/Ruhr (Germany) with Prof. M. T. Reetz. After post‐doctoral studies at Harvard University with Prof. E. N. Jacobsen, he started his independent career at the MPI of Molecular Physiology in Dortmund (Germany). Since 2007 he is full professor of Organic Chemistry at Graz University of Technology (Graz/Austria)*.